# Profound synchrony of age-specific incidence rates and tumor suppression for different cancer types as revealed by the multistage-senescence model of carcinogenesis

**DOI:** 10.18632/aging.203651

**Published:** 2021-10-25

**Authors:** Richard B. Richardson, Catalina V. Anghel, Dennis S. Deng

**Affiliations:** 1 Radiobiology and Health Branch, Canadian Nuclear Laboratories (CNL), Chalk River Laboratories, Chalk River, ON K0J 1J0, Canada; 2 Medical Physics Unit, Cedars Cancer Centre, McGill University Health Centre - Glen Site, Montreal, QC H4A 3J1, Canada; 3 Computational Techniques Branch, Canadian Nuclear Laboratories (CNL), Chalk River Laboratories, Chalk River, ON K0J 1J0, Canada

**Keywords:** aging, cancer incidence, cancer model, driver mutations, SEER program

## Abstract

The age-specific trend of cancer incidence rates, but not its magnitude, is well described employing the multistage theory of carcinogenesis by Armitage and Doll in combination with the senescence model of Pompei and Wilson. We derived empirical parameters of the multistage-senescence model from U.S. Surveillance, Epidemiology, and End Results (SEER) incidence data from 2000–2003 and 2010–2013 for The Cancer Genome Atlas (TCGA) cancer types. Under the assumption of a constant tumor-specific transition rate between stages, there is an extremely strong linear relationship (*P* < 0.0001) between the number of stages and the stage transition rate. The senescence tumor suppression factor for 20 non-reproductive cancers is remarkably consistent (0.0099±0.0005); however, five female reproductive cancers have significantly higher tumor suppression. The peak incidence rate for non-reproductive cancers occurs at a younger age for cancers with fewer stages and their carcinogenic stages are of longer duration. Driver gene mutations are shown to contribute on average only about a third of the carcinogenic stages of different tumor types. A tumor’s accumulated incidence, calculated using a two-variable (age, stage) model, is strongly associated with intrinsic cancer risk. During both early adulthood and senescence, the pace of tumor suppression appears to be synchronized across most cancer types, suggesting the presence of overlapping evolutionary processes.

## INTRODUCTION

Cancer incidence rates in adults generally rise exponentially with post-pubertal age, peak around 80 years, and then approach zero after age 100, although estimates are higher in centenarians if incidental tumors discovered at autopsy are included [1]. Mortality rates show a similar age-dependent trend delayed by survival, the time from clinical manifestation of the cancer until death [2, 3].

For most cancers in adults, the rise in the age-specific rate of cancer incidence *ASR*(*t*) can be represented by probabilities of occurrence of successive independent stages and by a power function [4]*.* The probability per unit time of the *i*^th^ stage occurring, up to a total of *k* stages can be denoted by *p_i_*. Using the notation and simplification described in the review by Frank [5], we assume that for each cancer, these probabilities are equal (*p*_1_*= p*_2_ *=…= p_k_ = u*). Therefore, if each stage of a unicellular or multicellular process proceeds at a small, roughly constant stage-transition rate *u* per year, the probability of any step occurring after *t* years is 1−e*^−ut^* ≈ *ut.* At age *t* the probability that *k*−1 steps have occurred is approximately (*ut*)*^k^*^−1^, and the final stage-transition rate is *u* (yr^-1^); therefore, the approximate rate (incidence) of occurrence at time *t* is

$$ASR(t)=(p_{1,}p_{2}\ldots p_{k})\;t^{k-1}=u^{k}\cdot \;t^{k-1}$$ 1)

Nordling [6] and Armitage and Doll [7] accounted for and modeled age-related cancer incidence as a power function that is dependent on a discrete number of ordered sequential stages:

$$ASR(t)=u^{k}\text{/}(k-1)!\;\cdot t^{k}{}^{-1}$$ 2)

Generalizing the factorial function to the gamma function as Γ(*k*) = (*k−*1)! for natural numbers *k*,

$$ASR(t)=u^{k}\text{/}\;\Gamma (k)\cdot t^{k-1}$$ 3)

To accommodate the old age decline in this multistage power function, Pompei and Wilson [8] developed their “beta model” of senescence, in which the empirical tumor suppression term (1−*bt*), with 0 ≤ *t* ≤ *b*^−1^, is based on the linear decline in cellular senescence or cell population doubling *b* (yr^-1^) and is equal to unity at birth for cancers of non-reproductive organs (henceforth referred to as “non-reproductive cancers*”*), or alternatively around the time of puberty, 15 years, for cancers of reproductive organs or “reproductive cancers*”*. Thus, *t* represents age for non-reproductive cancers, and (age − 15) ≥ 0 for reproductive cancers.

$$ASR(t)=a\cdot \;t^{k}{}^{-1}\cdot (1-bt),\text{ with }0\leq t\leq b^{-1}$$ 4)

Senescence tumor suppressor *b* (yr^-1^) is a constant parameter and *a* is a variable parameter that can be substituted according to Equation 3:

$$ASR(t)=\;u^{k}\text{/}\;\Gamma (k)\cdot t^{k-1}\cdot (1-bt)=A\cdot B\cdot C$$ 5)

where A is the “stage-transition-rate tumor suppression” term that is age-independent and exponential, *B* is an age-dependent “power law” growth term, and C is an age-dependent linear “senescence tumor suppression” term.

Harding et al. [9] produced best fit curves of the multistage-senescence model to U.S. Surveillance, Epidemiology, and End Results (SEER) program age-specific cancer incidence for three decades of data (1979–1983, 1989–1993, and 1999–2003). In a similar manner, we investigate the multistage-senescence model of carcinogenesis and model-fit to the age-specific rate of cancer incidence from SEER data sets for 2000–2003 and 2010–2013 [10] of The Cancer Genome Atlas (TCGA) cancer types [9, 11]. We show that just two variables (age and the number of carcinogenic stages) have a major influence, not only on the trend, but on the magnitude of age-specific incidence rate for all cancer types analyzed. In addition, the multistage-senescence model of carcinogenesis indicates that the number of stages is positively related to the age of peak incidence rate and inversely related to the stage-transition rate. Finally, the U.S. SEER data were matched to the cancer types from the National Cancer Institute TCGA data set to allow an estimate of the proportion of the total number of stages associated with driver gene mutations.

## RESULTS

The age-specific rate of cancer incidence was estimated following the methods of Harding et al. [9] from U.S. Census data [12] and the SEER cancer registry data for 2000–2003 and 2010–2013 [10] of 23 non-reproductive cancers as well as five female-specific cancers and two male-specific cancers. The parameters values are given in Table 1A–1C for geometric mean of the stage-transition rate *u*_μ_, senescence tumor suppressor *b*, and stages *k* for the weighted model-fits to SEER 2010–2013 incidence rates employing the multistage-senescence model (Equation 5) for males and females separately as well as for both sexes pooled. We will use the terms “both” or “both sexes pooled” to refer to the case when all incidence cases (both male and female, M&F) of non-reproductive cancer types were considered and corresponding estimates produced. We will use the term “male” when male-only cancer incidence data (both reproductive and non-reproductive) were tabulated and analyzed to produce estimates, and analogously for “female.” When we refer to “male and female,” the estimates were produced separately for males and females and the estimates (not the incidence data) were pooled. Exact probability *P* values are given unless the statistical test is insignificant when applying multiple testing correction.

**Table 1A:** Male (M) parameter values of model-fits to SEER 2010–2013 age-specific cancer incidence rates for reproductive and non-reproductive cancer types, employing the multistage-senescence model (Equation 5).

**Sex**	**Cancer type (TCGA)^0^**	**Model-fitted^1^**			**Peak incidence rate (per 100,000 person-year)**		**Age of peak incidence (yr)**		**Cumulative probability over lifespan**			**Ratio of cum. prob., SEER/2- variable**
		***u*_μ_ (yr^-1^)**	***k***	***b* (yr^-1^)**	**SEER**	**Model-fitted^2^**	**SEER**	**Model-fitted^3^**	**SEER**	**Model-fitted^4^**	**2-Variable model fitted**	
M	ACC	0.011	6.44	0.0107	0.347	0.301	87.5	79.2	0.00012	9.50E-05	0.010	0.011
M	BLCA	0.040	9	0.0098	344	380	92.5	91.0	0.10	0.10	0.017	6.1
M	COAD	0.029	6.9	0.0094	228	230	92.5	90.9	0.08	0.078	0.023	3.5
M	COADREAD	0.027	6.19	0.0092	274	279	92.5	91.2	0.10	0.11	0.026	3.9
M	ESCA	0.025	6.91	0.0101	42.5	47.4	82.5	84.9	0.016	0.015	0.014	1.1
M	GBM	0.019	6.16	0.0103	19.7	19.3	77.5	81.7	0.0067	0.0065	0.013	0.5
M	HNSC	0.019	5.07	0.0100	110	90.4	102.5	80.6	0.041	0.037	0.017	2.4
M	KICH	0.014	5.88	0.0106	4.13	3.65	72.5	78.2	0.0013	0.0012	0.011	0.12
M	KIRC	0.017	5.13	0.0105	41.9	37.9	72.5	76.9	0.014	0.014	0.013	1.0
M	KIRP	0.016	5.62	0.0106	13.5	11.4	72.5	77.2	0.0039	0.004	0.011	0.34
M	LAML	0.030	8.37	0.0098	42.0	45.5	87.5	89.5	0.014	0.013	0.016	0.85
M	LGG	9.20E-05	1.52	0.0085	0.502	0.363	82.5	40.3	0.0003	0.00030	0.032	0.0094
M	LIHC	0.015	4.57	0.0102	47.3	46.1	62.5	76.7	0.018	0.020	0.017	1.1
M	LUAD	0.035	7.81	0.0102	202	206	82.5	85.6	0.059	0.058	0.012	4.8
M	LUSC	0.035	8.4	0.0102	135	134	82.5	85.9	0.036	0.036	0.011	3.1
M	MESO	0.038	11.42	0.0100	11.0	12.7	92.5	91.5	0.0027	0.0027	0.014	0.19
M	PAAD	0.027	7.09	0.0101	74.9	75.7	82.5	85.1	0.023	0.023	0.014	1.7
M	PCPG	7.00E-06	1.33	-0.0168	0.407	NaN	92.5	-14.8	9.30E-05	NaN	NaN	NaN
M	READ	0.014	4.49	0.0089	51.0	49.9	97.5	87.8	0.023	0.025	0.031	0.73
M	SARC	0.015	6.4	0.0071	12.6	24.4	87.5	119	0.0042	0.012	0.13	0.031
M	SKCM	0.027	6.52	0.0093	220	195	102.5	91.0	0.075	0.070	0.024	3.1
M	STAD	0.026	7.24	0.0096	71.4	70.2	97.5	90.2	0.024	0.023	0.020	1.2
M	THCA	0.011	4.14	0.0103	19.8	18.3	72.5	73.6	0.0087	0.0082	0.017	0.52
M	THYM	0.012	6.11	0.0107	1.00	0.795	82.5	78.1	0.00028	0.00026	0.010	0.027
M	PRAD	0.035	5.11	0.0125	746	642	72.5	79.5	0.22	0.21	0.0055	40
M	TGCT	0.0013	1.52	0.0237	14.7	12.5	27.5	29.3	0.0040	0.0036	0.0067	0.60

^0^Non-reproductive cancers: ACC adrenocortical carcinoma, BLCA bladder urothelial carcinoma, COAD colon adenocarcinoma, COADREAD both COAD and READ cases, ESCA esophageal carcinoma, GBM glioblastoma multiforme, HNSC head and neck squamous cell carcinoma, KICH kidney chromophobe, KIRC kidney renal clear cell carcinoma, KIRP kidney renal papillary cell carcinoma, LAML acute myeloid leukemia, LGG brain lower grade glioma, LIHC liver hepatocellular carcinoma, LUAD lung adenocarcinoma, LUSC lung squamous cell carcinoma, MESO mesothelioma, PAAD pancreatic adenocarcinoma, PCPG pheochromocytoma and paraganglioma, READ rectum adenocarcinoma, SARC sarcoma, SKCM skin cutaneous melanoma, STAD stomach adenocarcinoma, THYM thymoma, THCA thyroid carcinoma.

Male reproductive cancers: PRAD prostate adenocarcinoma, TGCT testicular germ cell tumors.

Other abbreviations: NaN Not a Number.

^1^Equation 5; ^2^Equation 6; ^3^Equation 6 substituted in Equation 5; ^4^Equation 7.

The peak age-specific incidence rate, age of peak incidence rate, and cumulative probability of cancer over life span are computed up to maximum age 105, based on the SEER data alternatively model-fitted *ASR*(*t*). The age-specific incidence rate is computed per 100,000 population for each five-year age group. LGG and PCPG are cancer types with *P* values for model-fitted *u*_μ_ or *k* larger than 0.1 and were omitted from further analysis (see Supplementary Table 1A–1C).

**Table 1B:** Female (F) parameter values of model-fits to SEER 2010–2013 age-specific cancer incidence rates for reproductive and non-reproductive cancer types, employing the multistage-senescence model (Equation 5).

**Sex**	**Cancer type (TCGA)^0^**	**Model-fitted^1^**			**Peak incidence rate (per 100,000 person-year)**		**Age of peak incidence (yr)**		**Cumulative probability over lifespan**			**Ratio of cum. prob., SEER/2- variable**
		***u*_μ_ (yr^-1^)**	***k***	***b* (yr^-1^)**	**SEER**	**Model-fitted^2^**	**SEER**	**Model-fitted^3^**	**SEER**	**Model-fitted^4^**	**2-Variable model fitted**	
F	ACC	0.005	4.42	0.0105	0.396	0.343	77.5	73.7	0.00015	0.00014	0.0077	0.02
F	BLCA	0.030	8.08	0.0097	64.6	72.7	92.5	90.2	0.022	0.021	0.0071	3.1
F	COAD	0.030	7.23	0.0095	193	188	87.5	90.2	0.062	0.061	0.0086	7.2
F	COADREAD	0.028	6.55	0.0093	220	213	87.5	90.6	0.075	0.076	0.010	7.1
F	ESCA	0.020	7.09	0.0098	11.7	12.1	87.5	88.1	0.0043	0.0039	0.0075	0.58
F	GBM	0.015	5.56	0.0101	12.6	11.1	77.5	81.4	0.0042	0.0042	0.0075	0.56
F	HNSC	0.015	5.13	0.0092	31.4	29.5	92.5	87.1	0.013	0.013	0.013	1.0
F	KICH	0.0083	4.74	0.0104	2.21	1.86	77.5	75.6	0.00081	0.00075	0.0074	0.11
F	KIRC	0.013	4.63	0.0104	23.2	18	72.5	75.6	0.0073	0.0075	0.0078	0.94
F	KIRP	0.011	5.22	0.0105	3.56	2.98	77.5	77.3	0.0011	0.0011	0.0065	0.17
F	LAML	0.023	7.33	0.0097	22.0	23.1	82.5	88.8	0.0082	0.0072	0.0075	1.1
F	LGG	0.00093	2.65	0.0101	0.364	0.213	32.5	61.4	0.00022	0.00013	0.013	0.017
F	LIHC	0.018	6.06	0.0101	17.3	16.2	82.5	82.4	0.0057	0.0056	0.0067	0.85
F	LUAD	0.029	6.73	0.0101	162	144	77.5	84.0	0.047	0.046	0.0060	7.8
F	LUSC	0.028	7.65	0.0102	69.0	51.9	77.5	85.3	0.016	0.015	0.0051	3.1
F	MESO	0.018	7.85	0.0091	2.29	2.15	102.5	96.3	0.00065	0.00068	0.012	0.052
F	PAAD	0.027	7.18	0.0100	60.7	59.5	82.5	86.0	0.019	0.018	0.0062	3.0
F	PCPG	0.004	4.45	0.0109	0.182	0.111	72.5	71.1	6.10E-05	4.50E-05	0.0064	0.0094
F	READ	0.0043	2.82	0.0038	28.3	49.3	82.5	170	0.013	0.076	0.20	0.063
F	SARC	0.0093	5.01	0.0077	7.67	6.65	97.5	104	0.0023	0.0035	0.031	0.074
F	SKCM	0.012	4.08	0.0083	59.4	58.8	82.5	91.0	0.029	0.033	0.022	1.3
F	STAD	0.022	6.98	0.0094	34.4	33.1	102.5	91.2	0.012	0.011	0.0099	1.2
F	THCA	0.0053	2.36	0.0102	38.3	36.6	52.5	56.3	0.022	0.022	0.013	1.7
F	THYM	0.0087	5.34	0.0107	0.793	0.658	67.5	75.7	0.00025	0.00024	0.0056	0.045
F	BRCA	0.023	3.72	0.0115	435	428	77.5	78.5	0.19	0.18	0.0065	30
F	CESC	0.0011	1.57	0.0105	13.8	11.9	42.5	49.5	0.0071	0.0078	0.0094	0.76
F	OV	0.011	4.07	0.0121	12.7	11.9	72.5	77.5	0.0045	0.0046	0.0048	0.96
F	UCEC	0.013	3.4	0.0121	84.0	73.1	67.5	73.2	0.03	0.032	0.0061	5.0
F	UCS	0.010	5.59	0.0125	0.711	0.519	82.5	80.6	0.00018	0.00016	0.0022	0.081

^0^Female reproductive cancers: BRCA breast invasive carcinoma, CESC cervical squamous cell carcinoma and endocervical adenocarcinoma, OV ovarian serous cystadenocarcinoma, UCEC uterine corpus endometrial carcinoma, UCS uterine carcinosarcoma.

Abbreviations for non-reproductive cancer types and footnotes 1-4 are explained in Table 1A. LGG, PCPG and READ are cancer types with *P* values for model-fitted *u*_μ_ or *k* larger than 0.1 and were omitted from further analysis.

**Table 1C:** Both sexes pooled (M&F) parameter values of model-fits to SEER 2010–2013 age-specific cancer incidence rates for non-reproductive cancer types only, employing the multistage-senescence model (Equation 5).

**Sex**	**Cancer type (TCGA)^0^**	**Model-fitted^1^**			**Peak incidence rate (per 100,000 person-year)**		**Age of peak incidence (yr)**		**Cumulative probability over lifespan**			**Ratio of cum. prob., SEER/2- variable**
		***u*_μ_ (yr^-1^)**	***k***	***b* (yr^-1^)**	**SEER**	**Model-fitted^2^**	**SEER**	**Model-fitted^3^**	**SEER**	**Model-fitted^4^**	**2-Variable model fitted**	
M&F	ACC	0.0067	5.1	0.0105	0.329	0.319	77.5	76.6	0.00014	0.00012	0.0064	0.021
M&F	BLCA	0.036	8.47	0.0098	161	183	87.5	89.6	0.052	0.050	0.013	4.1
M&F	COAD	0.030	7.03	0.0095	203	203	87.5	90.1	0.068	0.067	0.014	5.0
M&F	COADREAD	0.027	6.32	0.0093	238	235	87.5	90.2	0.084	0.086	0.014	6.0
M&F	ESCA	0.022	6.64	0.0100	24.1	26	82.5	84.7	0.009	0.0085	0.0092	0.98
M&F	GBM	0.016	5.65	0.0101	15.7	14.3	77.5	81.6	0.0052	0.0053	0.0082	0.63
M&F	HNSC	0.017	4.87	0.0098	52.6	54.3	77.5	80.9	0.024	0.023	0.0088	2.7
M&F	KICH	0.010	5.17	0.0105	3.05	2.58	72.5	77.0	0.001	0.00098	0.0065	0.16
M&F	KIRC	0.014	4.69	0.0104	31.7	26.1	72.5	75.8	0.010	0.011	0.0066	1.5
M&F	KIRP	0.012	5.05	0.0105	8.04	6.33	72.5	76.4	0.0024	0.0024	0.0064	0.37
M&F	LAML	0.026	7.77	0.0098	29.6	31.4	82.5	88.8	0.010	0.0093	0.012	0.85
M&F	LGG	0.00064	2.34	0.0099	0.404	0.279	32.5	57.9	0.00026	0.00018	0.0026	0.10
M&F	LIHC	0.014	4.69	0.0102	28.4	28.7	77.5	77.5	0.011	0.012	0.0073	1.5
M&F	LUAD	0.030	7.07	0.0101	177	165	77.5	85.0	0.051	0.051	0.0091	5.7
M&F	LUSC	0.030	7.69	0.0101	95.8	81.4	77.5	85.8	0.024	0.023	0.0092	2.6
M&F	MESO	0.030	10.2	0.0099	5.09	5.69	87.5	90.8	0.0014	0.0013	0.014	0.10
M&F	PAAD	0.026	7.02	0.0100	66.5	65.7	82.5	85.5	0.020	0.021	0.0095	2.2
M&F	PCPG	0.0032	4.11	0.0103	0.188	0.133	92.5	73.4	7.30E-05	6.00E-05	0.0065	0.011
M&F	READ	0.011	4.12	0.0087	37.0	35.8	82.5	87.5	0.016	0.019	0.013	1.2
M&F	SARC	0.014	6.06	0.0084	8.74	8.96	97.5	98.9	0.0029	0.0037	0.025	0.12
M&F	SKCM	0.021	5.67	0.0094	107	107	82.5	87.9	0.045	0.043	0.013	3.6
M&F	STAD	0.024	7.02	0.0096	43.3	46.5	87.5	89.5	0.016	0.015	0.013	1.3
M&F	THCA	0.0059	2.69	0.0101	28.6	26.4	67.5	62.4	0.016	0.016	0.0042	3.7
M&F	THYM	0.0094	5.54	0.0106	0.747	0.705	67.5	77.0	0.00026	0.00025	0.0061	0.043
**M**	**Mean**	0.0231	6.8	0.0100	42.3	45.8	82.5	85.0	0.015	0.015	0.014	1.06
**M**	**SD**	0.0092	1.7	0.0008	95.9	99.6	10.7	9.8	0.030	0.029	0.027	1.73
**F**	**Mean**	0.0174	5.9	0.0098	22.6	20.6	82.5	85.6	0.0078	0.0073	0.0075	0.97
**F**	**SD**	0.0084	1.5	0.0008	52.1	49.4	11.8	10.0	0.016	0.016	0.062	2.22
**μ_M_, μ_F_^5^ *t* test, *P***		0.000056	0.0033	0.034^*^	0.014†	0.012†	0.45	0.32	0.012†	0.0094†	0.035*	0.73

^5^The mean, SD and paired *t*-test *P* values in each column are for the 20 non-reproductive, paired cancer types for males and females, for the parameter in the corresponding column.

†Values are no longer significant to 5% level when Holm’s method for multiple-testing correction is applied for the paired *t* tests in the table

*Values are no longer significant to 10% level when Holm’s method for multiple-testing correction is applied for the paired *t* tests in the table.

Male (M) and female (F) parameter values from Table 1A, 1B are compared by paired *t* test for 20 paired cancer types in the last row of the table.

^0^Abbreviations for non-reproductive cancer types and footnotes 1-4 are explained in Table 1A. LGG and PCPG are cancer types with *P* values for model-fitted *u*_μ_ or *k* larger than 0.1 and were omitted from further analysis.

Results from 2010–2013 are presented in this section and in Supplementary Table 1A–1C, whereas the similar results for 2000–2003 are found only in Supplementary Table 2A–2C. Unless stated otherwise, significant results hold for both the 2000–2003 and 2010–2013 data sets. Cancers having *P* values for model-fitted *u* or *k* larger than 0.1 were omitted from further analysis (Supplementary Table 1A–1C). Thus, for 2010–2013, glioma, LGG and neuroendocrine tumors, PCPG (TCGA abbreviations defined in Table 1A, 1B footnotes) were omitted from further analysis, as well as rectal cancer, READ for females. For the years 2000–2003, LGG and PCPG were omitted as well as adrenal cancer, ACC for females and both sexes, and sarcoma, SARC for females only. In addition, colorectal cancer COADREAD was omitted from some analyzes, such as from comparisons of *b* and *k* values, and from regression lines, when COAD and READ cases were included instead. Therefore, there are 20 and 19 paired cancer types for males and females for the 2010–2013 and 2000–2003 data, respectively.

### Senescence tumor suppressor

We fit the multistage-senescence model (Equation 5) to cancer incidence rate and analyzed the trends in the senescence tumor suppressor *b,* a parameter linked to declining cancer rates in very old age, with sex and different cancer types. There is insignificant variation (when adjusted for multiple testing) in *b* (yr^-1^) for non-reproductive cancers between sexes, according to the paired *t* test, with the *b* value for female cancers on average only about 2% smaller than that for males for the same cancer (*n* = 20, *P* > 0.1, Holm method). The mean *b* value for female non-reproductive cancers is 0.0098±0.0008 (SD, *n* = 20) and for males it is 0.0100±0.0008 (SD, *n* = 20, omitting rectal cancer, READ to compare paired values). The mean value of *b* for non-reproductive cases of both sexes pooled, omitting READ, is 0.0100±0.0005 (SD, *n* = 20), and 0.00991±0.0006 (SD, n = 21) including READ.

The mean value of *b* for all (non-reproductive and reproductive) male cancers is 0.0106±0.0030 (SD, *n* = 23, Table 1A, males) and for females 0.0102±0.0011 (SD, *n* = 25, Table 1B, females). We also performed multiple comparison tests, which are described in the Supplementary Note 1. The term (1 − *bt*) is zero when *t* = *b*^−1^ =98.2 years for females and 94.1 years for males.

The mean *b* value for female reproductive cancers is 0.0117±0.0008 (SD, *n* = 5, Table 1B, female). The *b* values of female reproductive cancers compared to the *b* values of their non-reproductive cancers (given above) are significantly different (*P* = 0.002), according to the Welch two-sample *t* test. This assessment was not repeated for male reproductive cancers as only two cancer types were analyzed (Table 1A, male).

### Stages, age of peak incidence, and stage-transition rate

We analyzed the trends in the number of cancer stages *k*, age of peak incidence (yr), and geometric mean of the stage-transition rate *u*_μ_ (yr^-1^) of the multistage-senescence model (Equation 5) with sex and different cancer types. There is a significantly greater (Welch two-sample non-paired *t* test, *P* = 0.019) mean number of stages of female non-reproductive cancers (*k* = 5.9±1.5 SD, *n* = 20) compared to their reproductive cancers (*k* = 3.7±1.4 SD, *n* = 5) (Table 1B, female).

**Figure 1:**
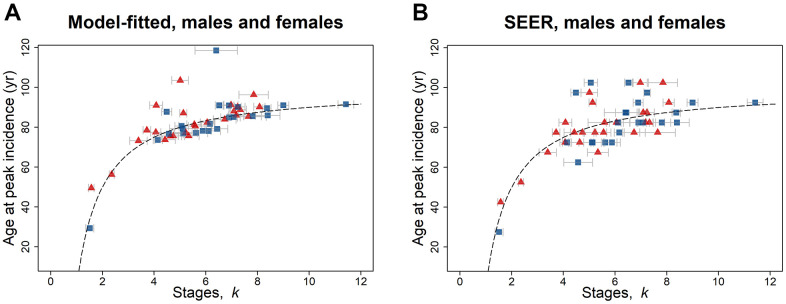
**Age at peak incidence (years) versus number of stages *k* for SEER data for 2010–2013, for males (blue squares) and females (red triangles) and for both reproductive and non-reproductive cancers (Table 1A, 1B).** Both (**A**) model-fitted and (**B**) SEER age at peak incidence have a highly significant positive association with *k*. The dashed line indicates the peak incidence age given by Equation 7 with *b* = 0.01. The gray bars indicate one standard error in the estimate of *k*.

The age of peak cancer incidence rate is dependent on the tumor suppression *b* and the number of stages *k* the cancer passes through until diagnosed; there is no dependence on geometric mean of the stage-transition rate *u*_μ_. By taking the derivative of Equation 4, the age of peak incidence rate can be calculated (Equation 7), and consequently the model-fitted peak incidence rate (per 100,000 person-year). The age at peak incidence rate increases in a non-linear manner with *k* for male and female cancers (Figure 1A, 1B). There is an extremely significant Spearman’s rank correlation with *k* for age at peak incidence rate, both model-fitted (both: Spearman’s *ρ* = 0.74, *P =* 0.0002, *n* = 21; female: *ρ* = 0.68, *P =* 0.0002, *n* = 25; male: *ρ* = 0.73, *P =* 0.0001, *n* = 23) and from the SEER data (both: *ρ* = 0.62, *P =* 0.003, *n* = 21; female: *ρ* = 0.66, *P =* 0.0003, *n* = 25; male: *ρ* = 0.47, *P =* 0.02, *n* = 23).

The model-fitted cumulative probability of being diagnosed with at least one of the cancers considered is 41% for females and 55% for males, computed using Equation 8. These values are similar to 42% for females and 56% for males calculated from the SEER data directly.

**Figure 2:**
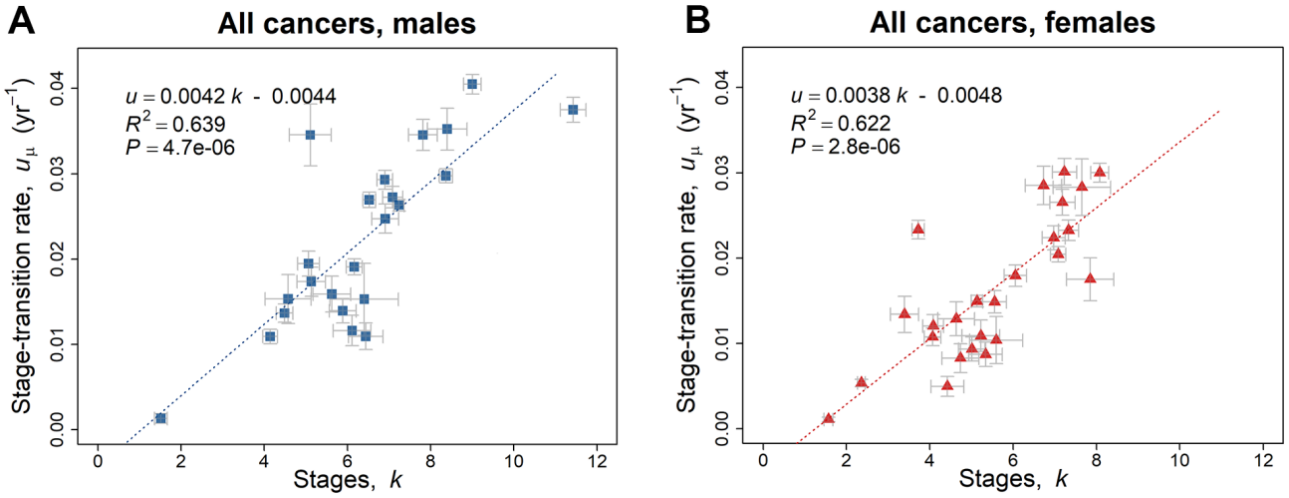
**Linear regression trend line of the geometric mean of the stage-transition rate *u_μ_* versus the number of stages *k*, employing the 2010–2013 SEER data based on the model of Equation 5.** (**A**) Blue squares represent values for males (Table 1A). (**B**) Red triangles represent values for females (Table 1B). The gray bars indicate one standard error in the estimate of the parameters by non-linear least squares.

There is an extremely significant linear relationship (*P* < 0.0001) of the geometric mean of the stage-transition rate *u*_μ_ with respect to the number of stages *k*, (Figure 2A, 2B). Given the assumption that stages occur at the same rate, Figure 2A, 2B indicate that the rate of change of each stage is faster when there are more stages in a cancer. However, if the assumption does not hold, one possibility that would be consistent with this pattern is that rates are slower during the initial stages of cancer initiation and increase during later stages (see Discussion). The linear regression analyzes of the geometric mean of the stage-transition rate *u*_μ_ versus the number of stages *k*, were compared for males and females by one-way analysis of variance, ANOVA and the slopes and intercepts did not differ significantly between paired regions (Supplementary Note 2).

There is an extremely significant variation in *u_μ_* for non-reproductive cancers between sexes, according to the paired *t* test, with the *u*_μ_ value for male cancers on average about one-third greater than that for females for the same cancer (*n* = 20, *P* < 0.0001).

### Two-variable model

In previous publications on the multistage and multistage-senescence models, only the trends in cancer incidence rate were analyzed, whereas we developed a two-variable model (age and the number of carcinogenic stages) that uniquely takes account of the relative magnitude of incidence rate of different cancers.

**Figure 3:**
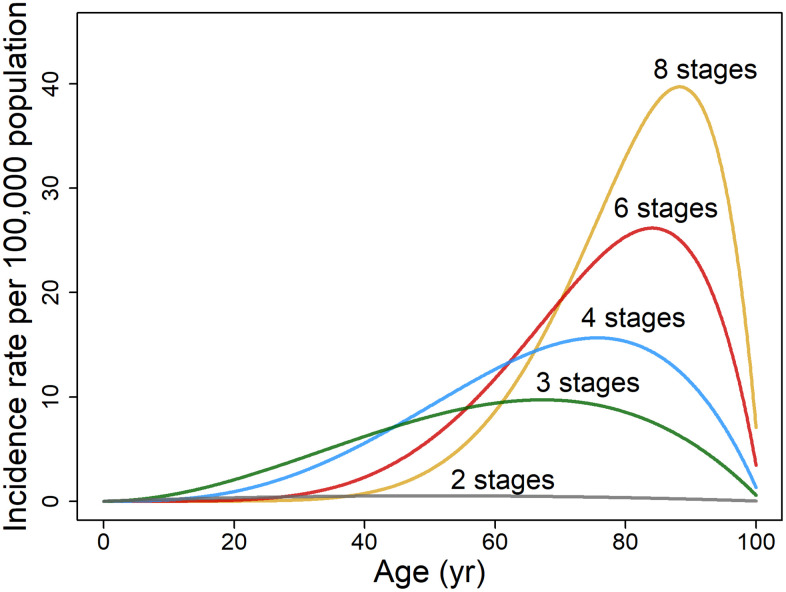
**Illustrative plots of age-specific rate of cancer incidence per 100,000 population based on two variables, age *t* and cancer stages *k*.** The age-dependent incidence rate of hypothetical cancers is modeled on non-reproductive cancers for both sexes pooled (*n* = 21) as described by Equation 6 (assuming *b* = 0.0099, *c* = 0.0046 and *d* = 0.0087).

An age- and stage-dependent model is obtained by substituting in Equation 5 for *u*:

$$ASR(t)={\left(c\cdot k-d\right)}^{k}\text{/}\;\Gamma (k)\cdot t^{k}{}^{-1}\cdot \left(1-bt\right)$$ 6)

where constants *b*, *c*, and *d* equal 0.0099, 0.0046, and 0.0087, respectively, as assessed for non-reproductive cancers of both sexes pooled (Figure 3). The effects of the senescence tumor suppressor factor employing the two-variable model are considerable as assessed from the increase in cumulative probability of cancer over a lifetime and the change in *b* value from 0.0099 (mean of non-reproductive cancers for both sexes pooled) to zero. For cancer stages *k* = 2, 3, 4, 6, and 8, the cumulative probability up to 101 years (=1/*b*) was reduced by 67, 75, 80, 85, and 88%, respectively (Supplementary Note 3), indicating greater tumor suppression *b* for the more complex cancers of longer latent periods. The age of

peak incidence rises from 67 to 76, 81, 87, and 90 years when *b* = 0.0099 as *k* increases from 2 to 3, 4, 6, and 8 (Equation 7). Complex cancers, compared to those with few stages, also have a higher cumulative incidence as is evident in the rise of probability over lifespan from 0.00034 to 0.0055, 0.0075, 0.0094, and 0.011, as *k* increases from 2 to 3, 4, 6, and 8.

There is an 1,800-fold range in the cumulative probability over lifespan of different cancers derived from SEER data (second-last column, Table 1A–1C), from 0.00012 for male adrenal cancer, ACC to 0.22 for prostate cancer, PRAD. The SEER to two-variable model ratios of the cumulative probability over lifespan were evaluated (last column, Table 1A–1C) and found to be the highest value of 40 for prostate cancer and 30 for breast cancer, BRCA, which have substantial environmental or extrinsic risk proportions [13]. When considering the non-reproductive cancers of either males or females, relatively high SEER to two-variable model ratios ranging from 3.2 to 7.8 are also found for bladder (BLCA), colorectal (COADREAD), lung (LUAD), skin (SKCM), and thyroid cancers (THCA), which correspond with cancer risk that is greatly influenced by extrinsic factors.

The ratios of known extrinsic to intrinsic incidence rates for 13 non-reproductive cancer types were obtained from data reported by Wu et al. [13], matching their cancer types to SEER TCGA cancers as closely as possible. The SEER to two-variable ratio of the cumulative probability over lifespan was strongly associated with the extrinsic to intrinsic cancer risk ratio (Spearman’s *ρ* = 0.73, *P* = 0.0047, *n* = 13).

### Driver mutations

In this subsection, the role of reported mutational cancer driver genes is quantified as a portion of the number of carcinogenic stages identified using the multistage-senescence model. The mean number of driver mutations as assessed by Iranzo et al. [14] for 20 TGCA non-reproductive cancers (excluding glioma, LGG and neuroendocrine tumors, PCPG) of both sexes pooled was 1.7 compared to 6.2 stages obtained by best fit to the multistage-senescence model (Table 1C). Therefore, on average about two-thirds of the cancer stages do not involve driver genes. This premise assumes that the multi-stage model is valid, and that each driver mutation contributes to a single stage, which may not be the case (see Discussion).

**Figure 4:**
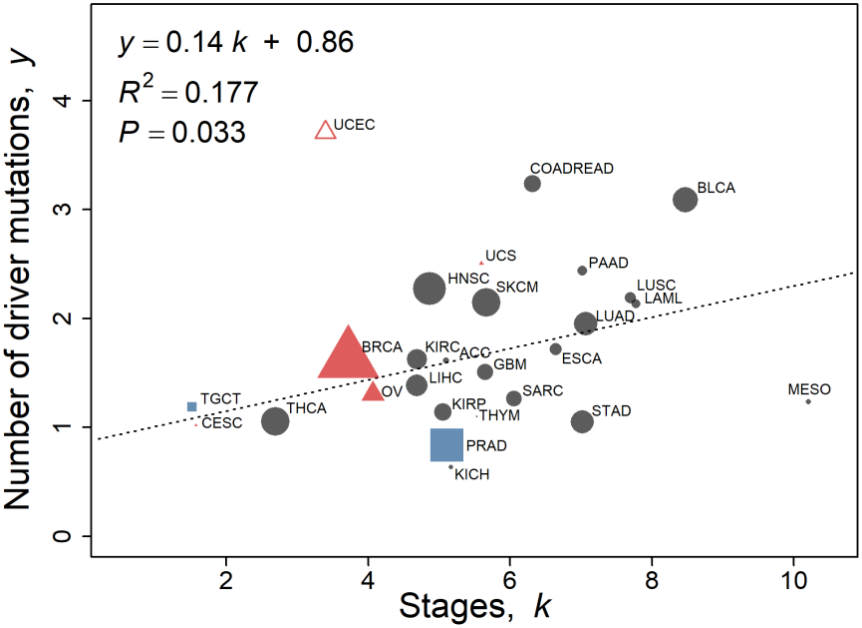
**Number of driver mutations as assessed by Iranzo et al. [14] with respect to stage *k* calculated using the multistage-beta model employing the 2010–2013 SEER data.** Values of non-reproductive cancers for both sexes pooled (Table 1C, *n* = 20) are represented by black circles. Female-specific cancers BRCA, CESC, OV, UCEC, and UCS are represented by red triangles, and male specific cancers PRAD and TGCT are represented by blue squares. UCEC is omitted as an outlier (standardized residual ≥3.0). The linear regression fit to the data is *y* = 0.14 *k* + 0.86, where *y* is the number of driver mutations as assessed by Iranzo et al. [14]. The regression is not weighted. The size of the points is proportional to the number of TCGA cases of that cancer type for illustration only.

We investigate whether the number of stages *k* is related to published evaluation of the number of driver genes for various cancer types using the 2010–2013 SEER data (Figure 4). The outlier endometrial cancer, UCEC (standardized residual ≥3.0), was excluded from the analysis as it was the only type of cancer that demonstrated more driver genes than the number of stages. Endometrial cancers consist of four categories with highly variable mutation frequency and copy number [15]. Driver mutations from Iranzo et al. [14] are linearly correlated to a moderate degree with the number of stages in the 2010–2013 data (Pearson’s *r* = 0.42, *P* = 0.033, *n* = 26) and in the 2000–2003 data (Pearson’s *r* = 0.39, *P* = 0.054, *n* = 25). In terms of percentage contribution from driver mutations to the number of stages, the mean is 34% (similar in value to that derived from Table 2) with a wide 95% confidence interval of 9.9% and 64.3%, possibly partly due to differing contributions of driver genes to carcinogenesis as detailed in the Discussion. Comparing the number of stages with the number of driver genes from Bailey et al. [16] yields a weak correlation (Pearson’s *r* = 0.31, *P* = 0.12, *n* = 26 from 2010–2013 data; Pearson’s *r* = 0.30, *P* = 0.15, *n* = 25, from 2000–2003 data).

**Table 2:** The number of driver genes and mutations as assessed by Iranzo et al. [14] and Bailey et al. [16], compared to the number of stages as given for both sexes of SEER incidence data pooled (Table 1C), except for reproductive cancers (Table 1A, 1B).

**TCGA**	**Driver genes mutations (Iranzo et al.)**	**Driver genes mutations (Bailey et al.)**	***SEER k* stages**	**Non-driver gene stages (Iranzo et al.)**	**Non-driver gene stages (Bailey et al.)**
ACC	1.61	0.52	5.10	3.48	4.58
BLCA	3.09	5.10	8.47	5.38	3.37
BRCA	1.62	1.84	3.72	2.10	1.88
CESC	1.02	1.89	1.57	0.55	-0.32
COADREAD	3.24	3.84	6.32	3.09	2.48
ESCA	1.72	1.87	6.64	4.92	4.77
GBM	1.51	1.84	5.65	4.15	3.81
HNSC	2.27	3.21	4.87	2.59	1.66
KICH	0.64	0.48	5.17	4.53	4.69
KIRC	1.63	1.45	4.69	3.06	3.24
KIRP	1.14	0.35	5.05	3.91	4.70
LAML	2.13	0.77	7.77	5.64	7.00
LIHC	1.38	1.84	4.69	3.30	2.85
LUAD	1.95	2.19	7.07	5.12	4.88
LUSC	2.19	2.68	7.69	5.50	5.01
MESO	1.24	0.87	10.2	8.97	9.33
OV	1.31	1.19	4.07	2.76	2.88
PAAD	2.44	2.19	7.02	4.58	4.83
PRAD	0.84	0.55	5.11	4.27	4.56
SARC	1.26	0.63	6.06	4.79	5.43
SKCM	2.15	2.45	5.67	3.52	3.22
STAD	1.05	1.92	7.02	5.97	5.10
TGCT	1.19	0.34	1.52	0.33	1.18
THCA	1.06	0.77	2.69	1.64	1.92
THYM	1.10	0.63	5.54	4.44	4.91
UCEC	3.71	7.34	3.40	-0.31	-3.94
UCS	2.51	3.13	5.59	3.09	2.46
Average [including UCEC]Average excluding UCEC	[1.74] 1.67	[1.92] 1.71	[5.49] 5.58	[3.75] 3.91	[3.57] 3.86
Average (non-reproductive only)	1.74	1.78	6.17	4.43	4.39

## DISCUSSION

This analysis shows that the synchrony in both the rise and fall of U.S. SEER cancer incidence in aging adults is profound, with the possible evolution of the stage-transition rate and senescence tumor suppression mechanisms. However, the task of disentangling and quantitatively identifying aging effects on cancer etiology is recognized to be especially difficult, with on the one hand effects that promote cancer such as inflammation and genomic instability [17] and on the other hand senescence-related defensive systems that suppress cancer.

### Senescence tumor suppression

The tumor suppression factor *b* (yr^-1^) is relatively constant for non-reproductive cancers regardless of cancer type or sex. Senescence tumor suppression is assumed to increase linearly with age. There are various potential cellular mechanisms that likely contribute to the suppression of malignant cancers in old age such as telomere erosion, Hayflick limit, stem cell exhaustion, senescent cells, accelerating systemic mass loss, and epigenetic aging changes [18–20]. For example, apoptosis rates in bone marrow increased from ~7% of cells, both in 0- to 9-year-olds and in 50- to 59-year-olds, rising to three-fold more in 80- to 100-year-olds [21]. The tumor suppressor factor is of a similar value whether the malignancy is mesothelioma primary arising from inhaled asbestos, cervical cancer from exposure to human papillomavirus, melanomas from sun exposure, or post-menopausal breast cancer with elevated insulin levels. The constancy of the tumor suppression factor indicates tissue adaptation to various extrinsic environmental causes of cancer. The suppression of malignant tumors is accompanied by an increase in the prevalence of benign cancers. Imaida et al. [22] studied autopsies of 871 Japanese patients aged 48 to 113 years at death and found that the ratio of prevalence of latent cancers (those not diagnosed clinically) to cancers with metastasis increases from 0.64 in 48- to 84-year-old patients to 1.4 in older patients. Although there is evidence of mosaic aging of normal tissues [23], overwhelmingly there is synchrony in systemic aging [24] and the tumor suppressor factor, which is accompanied by the decline of cancer incidence rates for all cancer types in the aging adult.

The aggregated SEER cumulative probability over lifespan of 20 non-reproductive cancers analyzed is 73% greater for males than for females (Table 1C). In parallel with this, males have a greater acceleration of mass loss than females as measured in some major organs and body cell mass of normal populations [24]. This may be indicative of a greater rate of aging in males [25]. DNA mutation accumulation is greater in sex-specific cancers [26]. The tumor suppression of female reproductive cancers is shown to be stronger than for female non-reproductive cancers. A possible tumor suppressive mechanism is the early onset of mass loss in female reproductive organs compared to non-reproductive ones (breast, ovary, and uterus at about 25, 35, and 21 years of age, respectively) and an elevated decline in mass loss of functional tissue (breast, ovary, and uterus lose about 35, 46, and 35% mass, respectively, from 25 to 70 years of age) [24]. This greater suppression of female reproductive cancers may be an evolutionary adaptation to counter estrogen having a strong proliferative effect.

The interplay between the p53 master tumor suppressor and insulin-like growth factor *1* (IGF-1), which stimulates the mammalian target of rapamycin (mTOR), is critical to normal cell growth and carcinogenesis: viable p53 down-regulates these two highly evolutionary-conserved pathways [27]. In fact, insulin, growth factors and amino acids all activate the mTOR pathway, which stimulates protein synthesis and cell growth [25]. IGF-1 production provides an important protein determining post-natal growth and growth hormone (GH) signaling. Adversely, IGF-1 is not directly mutagenic but a potent mitogen and cancer risk; for example, prospective blood samples show elevated IGF-1 levels in individuals later diagnosed with prostate and pancreatic cancer [28, 29]. IGF-1 has also been implicated in increased cancer risk of breast, colorectal, lung, and other cancers [30]. The down-regulation of the GH/IGF-1/insulin system decreases cancer risk and increases longevity in animal models; however, in humans the results are somewhat contradictory, although genetic studies of the GH/IGF-1/insulin system support their involvement in human longevity [31]. Cancer is virtually unknown in patients with congenital IGF-1 deficiency, exhibiting dwarfism and obesity [32]. A meta-analysis study confirms that the prevalence of most cancers increases with adult height, which is influenced by hormone levels, especially growth factors [33]. Multiple studies of serum IGF-1 concentrations show an increase in adolescence, a peak during puberty, and then initially in adulthood a rapid decline; thereafter IGF-1 levels change more slowly to about a fifth to a tenth of the maximal value at 80 years of age [34, 35]. For both sexes, the multicenter and largest study [36] measured the annual fractional decline in IGF-1 activity (estimated as the gradient over the intercept of a linear trend between 25 and 80 years) of about 0.0087, which is similar to the tumor suppressor factor *b* value of 0.0099. Growth hormones can have a profound influence on both normal and carcinogenic tissues. Organ functional mass loss, greater than fat-free mass loss, starting early in adulthood and accelerating in old age may be indicative of the reduction in primary growth hormones and metabolic rate with adult age [24, 37]. Consequently, the systemic cancer suppressor factor *b* and involutional changes could therefore be associated with the declining plasma IGF-1 in aging adults.

Cancer and aging are inextricably interconnected. In 1957, Williams [38] proposed the theory of antagonistic pleiotropy: genes such as *TP53* or transforming growth factor*-β* (*TGF-β*), and biological processes that enhance reproductive success early in life, lead to an evolutionary trade-off, with later fitness decline and death. In this view, p53-dependent replicative senescence would be one such biological process. In terms of cancer, p53, replicative senescence, and indeed growth hormones are a double-edged mechanism: they can both advance and impede oncogenesis [39, 40]. Contemporary observations are that longevity-enhancing, protective, genetic variants become more prevalent with increasing age of the very old [41]. The heritability of living to the mid-80s is only 20–30% (twin study [42]; however, the heritability of living past 100 is between 33% (females) and 48% (males). Hence, there is the possibility that the evolution of tumor suppression, although associated with frailty, nevertheless counteracts cancer in the very old.

### Stages

This work demonstrates that adult cancers with an early age of peak incidence rate (yr) have fewer cancer stages *k* than more complex cancers that reach maximum incidence rate at a later age (Figure 1A, 1B). The multistage model is based on cellular changes that are specific, discrete, and stable and that proceed in a unique order, although the changes are not necessarily gene mutations [7, 43]. Malignant tumors typically acquire a range of biological capabilities or hallmarks that include proliferating in a sustained manner, evading growth suppression, resisting cell death, acquiring replicative immortality, inducing angiogenesis, and activating invasion and metastasis [17]. Biological mechanisms that support these hallmarks include genetic mutations and epigenetic modifications [5, 44]. Most cancer cell lines also exhibit very short telomeres but escape replicative senescence through mechanisms such as telomerase activation or telomeric recombination [45–47]. Four-fifths of tumors are solid tumors, which generate a blood vascular supply to supply nutrients and oxygen to enable growth beyond a few millimeters. Two-thirds of solid tumours in a Norwegian population registered with metastases at death [48]. This process involves genetic and epigenetic changes in which cells commonly change their phenotype such as during epithelial-to-mesenchymal transition or mesenchymal-to-epithelial transition and exhibit hybrid features via intermediate or partial states [49, 50].

### Stage-transition rate

The geometric mean of the stage-transition rate *u*_μ_ (yr^-1^) has a wide range from 0.00064 to 0.040 for the various cancer types (Table 1A–1C) and its increase with the number of stages *k* is remarkably robust (Figure 2A, 2B). For example, testicular cancer, TGCT with 1.5 stages has a value of *u_μ_* of 0.0013, whereas for bladder cancer, BLCA with 8.5 stages, *u*_μ_ is 0.036 for both sexes pooled. Therefore, if the number of cancer stages is small, the duration of each stage is longer. This finding may result from earlier stages being of longer duration than later ones. One could speculate that if, contrary to our findings, the transition rate was initially rapid, then the cumulative incidence of the cancer could be extremely high by middle age, so there would be strong evolutionary pressures to reduce the incidence of the cancer. This pressure could result in addition obstacles to the cancer's formation, which would then require additional mutations for a cancer to overcome. In this way, a fewer-stage cancer could be converted to a many-stage cancer. Therefore, it appears that cancer cells out-compete aging, slower-dividing, normal cells by acquiring quickening carcinogenic stages via Darwinian selection that widens the cancer cell traits from those of the initial cell, and in the process augments the competitive advantages of cancer cells (especially high-stage tumors) over those of the surrounding non-cancer cells [51].

Several tumor suppression factors have been posited as influencing the generally lower cancer rates in women than men, including adult females having generally shorter stature, longer telomeres, less telomere attrition and lower rates of thymic involution, healthier lifestyles, better T cell production, and more robust p53 response [52–54]. An exception is DNA methylation, which is the most accurate parameter of biological aging and a tumor suppression factor that has equal influence in men and women [20]. We identified some additional gender-specific carcinogenic factors by comparing the parameters of the multistage-senescence model derived for 20 types of non-reproductive cancers in males and females (Table 1C). For example, cancers in males have ~14% more stages than those in females (*P* = 0.0033). Intriguingly, the geometric mean of the stage-transition rate *u*_μ_ in males is 33% greater than that of females (*P* < 0.0001). This is a novel explanation of male susceptibility to carcinogenesis. However, it is worth further consideration because a small change in the stage-transition rate *u* results in a large change in the incidence rate, as *u* is raised to the power *k*. Surprisingly, there is no sex-dependent significant difference in the extrinsic-risk-dependent parameter, the SEER to two-variable model ratio of the cumulative probability over a lifetime, which tentatively infers that male susceptibility is not due to lifestyle.

### Two-variable model

Although the two-variable model based on age- and stage-dependence is too broad a stroke to characterize cancer incidence for all cancer types, it is instructive to analyze the trends it describes. The two-variable model for non-reproductive cancers indicates that for a small number of stages, the peak incidence rate is lower and occurs at a younger age (Figure 3). Complex cancers, compared to those with few stages, have a much higher cumulative incidence; for example, the cumulative probability over lifespan of an eight-stage cancer is 33 times that of a rarer two-stage cancer. Therefore, in general, the more complex the staging of specific adult cancers, the higher the cancer incidence rates and the longer the latent periods. Another observation is that the early age of peak incidence rates of cancers with fewer stages have far broader incidence rate peaks than the more complex cancers, somewhat due to lesser influence of senescence earlier in adulthood. Age 60 years appears pivotal; before that age cancers with few stages dominate diagnoses, whereas after that age high-stage cancers dominate incidence rates.

There is great variation in the endogenous (e.g., biologic aging) and exogenous (i.e., radiation) non-intrinsic factors driving the total cancer risk and the age-adjusted incidence rate of regions around the world, with the ratio of high to low incidence rates being up to ten-fold or more [13]. A finding was that the two-variable, age- and stage-dependent model is highly dependent on the intrinsic risk of carcinogenesis [13]. This was determined by the strong association (*P* = 0.005) of the ratio of the cumulative probability of cancer over lifespan, calculated from SEER data (numerator) and the two-variable model (dominator), with the ratio of the extrinsic cancer risk (numerator) and the intrinsic cancer risk (dominator). Hence, there appears to be an evolutionary component to multistage [55] and multistage-senescence models, where natural selection acts to suppress early-onset, rare, low-stage cancers when able to effect Darwinian fitness, which is less effective as reproductive rates decline and late-onset, common, high-stage cancers develop.

### Driver mutations

All cancers possess somatically acquired mutations. Most somatic mutations are passenger mutations, which far outnumber, and rise with, the number of mutational driver genes [14]. Sporadic cancers arise through somatic evolution that parallels the increase in mutations in normal cells with aging. Early events in a cancer’s development are characterized by a constrained set of common driver genes, and later events are ascribed to a greater set (~four-fold more) of drivers and increased genomic instability [56]. This applies to different cancer types and subtypes. Primary tumor cells may lie dormant for years before circulating cells form metastases [50]. Only ~0.03% of circulating melanoma cells in mice formed lung metastases [57]. However, other researchers report that the formation of driver gene mutations mainly arises during the initial stages of carcinogenesis, as primary and metastatic tumors share almost the same driver genes [58]. Non-shared metastatic driver genes do not have functional consequences. The median number of mutated driver genes was three for adult acute myeloid leukaemia, with the number of driver mutations increasing with age [59]. The mean number of driver gene mutations of various cancer types is approximately two (Table 2), [14, 16].

Mutations in the *TP53* gene (or p53 protein), the most common driver gene in cancers, particularly epithelial cancers, significantly increase in tumors with a high number of stages as evaluated from the steepness of the power function rise in cancer rates with aging (Equation 1) [60]. Driver gene mutations can contribute to one or more stages. This quantitative trend of the loss of the *TP53* gene increasing with a cancer’s stages can be interpreted as p53 contributing to multiple carcinogenic stages and promoting increasingly rapid progression. Early mutations dominate cancerous tissues, as shown in breast cancer [61]. This is supportive of metastatic driver genes arising early in the cancer development, (especially those involving oncogenes *TP53* and *KRAS)* [62], although further stages comprise the metastatic process. Notwithstanding the central dogma in oncology of the dominant role of driver genes and mutations, by our analysis driver mutations contribute (making no allowance for non-singular driver mutations contributions) to only approximately one-third of the carcinogenic stages, which underlines the complexity of tumorigenesis [63].

### Models of carcinogenesis

There are many alternatives to the multistage-senescence model of carcinogenesis. A review of carcinogenic models identified five mathematical model types, namely mutations, genomic instability, non-genotoxic mechanisms, Darwinian cell selection, and tissue organization [29]. A well-known model by Moolgavkar, Venzon, and Knudson (MVK) [64], based on a two-mutation initiation-promotion process, seeks to address a considered deficiency of the Armitage–Doll model [7] in that it takes account of cell division and differentiation. This model is especially suited to childhood cancers such as the most common type, acute lymphoblastic leukemia, and the much rarer tumor, retinoblastoma, which have been experimentally and theoretically shown to have undergone two genetic changes [65, 66]. Childhood cancers often result from defects in developmental signaling pathways of stem cells [19, 67]. Obviously, the primary incidence peak of childhood cancers is not influenced by senescence as represented by the multistage-senescence model. However, cancer models have been produced based on genomic instability and the somatic cellular evolution of cancer likely common to tumors of all ages [68]; notwithstanding, childhood and adult cancer develop and require models of a different nature.

The decline in cancer incidence rates in old age has been modeled by Cook et al. [69] for stomach cancer in males assuming that only 1% of the population is susceptible to this cancer; the modeling led to similar fits between the susceptibility and multistage-senescence models except for those aged more than 105 years. Our model is in agreement with the analysis of Cook et al. [69], indicating incidence peaks at a younger age for rarer cancers. However, Ritter et al. [70] suggest that it is unlikely that cancer-susceptible people are depleted after age 80. A recent analysis by Belikov [71] shows that Erlang probability distributions (summing independent, exponentially-distributed events or stages) closely follow U.S. SEER incidence rate curves for 20 prevalent cancers. No account is taken of a cellular-senescence component being a factor in cancer downturn in old age. The analysis generates the number of successive, carcinogenic events, with a very wide and perhaps improbably large range of 4 to 41 stages, proffered to be driver mutations or epimutations. Based particularly on colorectal cancer common in adulthood, it has been proposed that three driver genes are needed for a cell to evolve through breakthrough, expansion, and invasive stages to an advanced cancer [72]. Whereas our study estimates the number of stages for colorectal cancer, COADREAD (Table 1A–1C) as between six and seven stages, maybe including observed epigenetic modifications [5, 44]. The Armitage–Doll and MVK models both assumed a constancy in the number of stages for all tumor types, although analysis of contemporary SEER and driver gene data and contemporary understanding of the development of genomic instability, epigenetic changes, and metastases indicate that this is certainly not the case.

### Limitations of determining temporal trends in cancer incidence

Finally, there is a limited potential to identify temporal trends in multistage-senescence model parameter values as seen in the five data sets consisting of the 1979–1983, 1989–1993, and 1999–2003 data from Harding et al. [9], and the corresponding 2000–2003 and 2010–2013 data from Supplementary Tables 5A–5C, 4A–4C, respectively. For example, a fall in the number of stages of prostate cancers from ten to about five was a trend evident in the SEER data sets from 1979–1983 to 2010–2013 (Supplementary Figure 1). Care was taken to duplicate the data retrieval and fitting methods of Harding et al. [9], as three of the five data sets originate from this published study. Of course, ideally all data sets used would derive from our labors, although data from the 2000–2003 and 2010–2013 groups come from a larger geographic region than those of earlier years. There is an overlap in the 1999–2003 and 2000–2003 data sets, with four independent data sets, not five, which limits temporal analyzes.

The prostate cancer incidence rate is influenced by its detection by transurethral resection of the prostate and by prostate-specific antigen (PSA) [73]. The use of PSA tests from 1986 onwards in the U.S. has allowed the early detection of prostate cancer. This has also resulted in considerable overdiagnosis because detection of prostate cancer would otherwise not have been diagnosed within the patient’s lifetime. An Australian study indicated that overdiagnosis was common in prostate, breast, renal, and thyroid cancers, and melanoma [74]. An analysis of SEER data from 1988 to 1998 indicated over-diagnosed prostate cancer rates of ~29% for white subjects and 44% for black subjects [73]. It is proposed that early- and over-diagnosis due to PSA availability may partly explain the declining number of stages in prostate cancers diagnosed over four decades. This demonstrates the potential to examine temporal trends in incidence-related parameters, provided there is future analysis of more data sets.

A generalized limitation of our analyzes of age-specific rate of cancer incidence is their cross-sectional nature. The age-specific incidences rates emanate from various groups of people, who may have different cancer risks for reasons other than age itself, and these reasons are not accounted for in the carcinogenesis models. Hanson et al. [75] point out that cross-sectional cancer incidence studies often offer limited conclusions about cancer trends due to aggregated ages above 85 years or the examination of a single period or fail to consider period and cohort influences — only the last of these is a limitation of our study. There are significant differences between the characteristics of the TCGA and SEER datasets, such as age and stage at diagnosis [76]. The potential inaccuracy of population estimates for age groups above 85 years was investigated in a data quality study by Miller et al. [77] that analyzed (in a similar manner to our study) the 2010 Census and U.S. SEER registry records for 2008–2012, which yielded cancer incidence rates that usually did peak (a trend observed in our study) and then decrease in the oldest old.

## CONCLUSION

A multitude of carcinogenic models have been proposed in the scientific literature; however, the multistage-senescence model is unique in quantifying several important parameters of the age-specific incidence rate of different cancer types based solely on the number of development stages. The finding, perhaps controversial, that cancer-suppressive mechanisms counter the aging-dependent escalation in cancer complexity and incidence gives support to preventing cancer by augmenting extant mechanisms. The two-variable model gives us a useful tool to further investigate the biological mechanisms that drive specific cancers, such as those that are affected by ethnicity or ionizing radiation.

## MATERIALS AND METHODS

All statistical analysis was performed using software R version 3.6.0 [78]. The full code for all analyzes (including processing data, computing incidence rates, statistical analysis, and making plots) that we developed at the time of publication, under MIT License, for estimating cancer incidence rates is posted in the GitHub repository at https://github.com/canghel/cancer-incidence-v5.

### SEER cancer incidence data

The methods to estimate incidence rates from person-years at risk for the older U.S population and to fit the multistage-senescence model were reproduced as closely as possible from Harding et al. [9]. The age-specific rate of cancer incidence data from the SEER cancer registry were analyzed were for 2000–2003 and 2010–2013. This was to allow use of the SEER 18 registries data for all the calculations [10], to be near in date to the Census 2000 and 2010 population estimates [12], and to allow reasonable comparison to previous literature. As in Harding et al. [9], we restricted the selection of cases to malignant behavior, known age, and first matching record for each person.

The incidence cases of 30 cancer types corresponding to TCGA cancer types [76] were extracted from the SEER data [10] using SEER*Stat 8.3.5 software. The cancer types consist of 23 non-reproductive cancers as well as five female-specific cancers and two male-specific cancers. The TCGA codes and SEER histology correspondence are given in Supplementary Table 3 [76]. The number of cancer cases of each type, sex, and age-range was downloaded separately for each of the 18 SEER registries.

For ages up to 85 years the SEER-provided population estimates were used. The U.S. Census 2000 and 2010 populations [12] for each registry agree well with the 2000 and 2010 corresponding SEER populations for all registries except for the Alaska Native Tumor Registry. For ages 85 and greater we calculated the fraction of the 85+ population by sex in each registry and in each of the age groups 85–89, 90–94, 100–104, 105–109, and 110+ using the U.S. Census data for 2000 and 2010. We used the Census population older ages fractions to infer the populations for the oldest categories, assuming that these fractions remain constant over the subsequent three years. The discrepancy between such ratios is less than ~15% for the groups under 85 years.

The crude rate per 100,000 persons in each five-year age category were determined and the associated standard error. Although the assumption of constant 85+ population fractions over time may fail for the oldest populations, especially for males with higher mortality rates, the computed standard errors for the incidence rates in these populations are also large and hence these incidence rates to the multistage beta model fit. For the peak age of incidence and the peak rate of incidence, we restricted the values reported to ages less than 105 to avoid outliers.

### Multistage-senescence model fit

We fit the multistage-senescence model (Equation 4) by non-linear least squares, weighted proportionally to the inverse standard error squared, using the Levenberg–Marquardt algorithm from the minpack.lm v.1.2-1 package [79] in R v.3.6.0 [78]. The curves were fit beginning at age 50 when the number of cases for each cancer begins to rise [9], with three exceptions where the incidence peaks at much younger ages than most cancer types: thyroid carcinoma (THCA) and cervical squamous cell carcinoma and endocervical adenocarcinoma (CESC) both for females at 30 years, and testicular germ cell tumors (TGCT) in males at 20 years. The time *t* in Equations 4 and 5 corresponds to age in non-reproductive cancers and to time since the onset of puberty, estimated as age minus 15 years in reproductive cancers.

By taking the derivative of Equation 4 [80], the age of peak incidence is

$$T_{p}(k)=k/(b\cdot (k+1))$$ 7)

The total probability of each cancer type, the cumulative ASR, computed analogously for Equation 4 as in Pompei and Wilson [80] is

$$\begin{array}{c} P_{c}=\int_{0}^{1/b}ASR(t)\text{d}t=\int_{0}^{1/b}at^{k-1}(1-bt)\text{d}t\\ =\;\frac{a}{b^{k}}\left(\frac{1}{k}-\frac{1}{k+1}\right)=\;\frac{u^{k}}{\Gamma (k)}\frac{1}{b^{k}}\left(\frac{1}{k}-\frac{1}{k+1}\right) \end{array}$$ 8)

Parameter *a* is a multiplicative term [7], whose values are given in Supplementary Tables 4A–4C, 5A–5C.

### TCGA driver gene data

The number of driver mutations are taken from Table S6, of Iranzo, Martincorena [14], with the total number of samples per cancer type assumed to be the same as in Martincorena, Raine [81].

### Additional information

Supplementary information is available in the online version of the paper. The full code and additional documentation at the time of publication, under MIT License, is posted in the GitHub repository at https://github.com/canghel/cancer-incidence-v5.

## Supplementary Material

Supplementary Figure 1

Supplementary Tables

Supplementary Notes
