# Supplementary material for: Profound synchrony of age-specific incidence rates and tumor suppression for different cancer types as revealed by the multistage-senescence model of carcinogenesis
Source: Aging (Albany NY). 2021 Oct 25;13(20):23545–78. doi: 10.18632/aging.203651 (PMC8580351; doi:10.18632/aging.203651)
Supplement: Supplementary Figure 1 [file aging-13-203651-s001.pdf]

SUPPLEMENTARY FIGURE

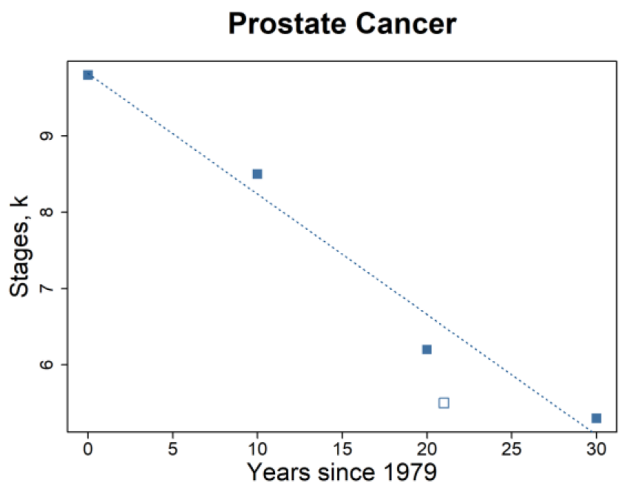

Supplementary Figure 1. Stage *k* for prostate cancer versus timeline for SEER data from the 1979–1983, 1989–1993, and 1999-2003 studies (Harding, Pompei et al. 2008) and the corresponding data for 2010–2013 from Supplementary Table 4A. Data for 2000-2003 is shown as an outlined square (Supplementary Table 5A), for comparison with 1999-2003 data, but is not included in the regression line fits due to the overlap in data sets.
