# Supplementary material for: Profound synchrony of age-specific incidence rates and tumor suppression for different cancer types as revealed by the multistage-senescence model of carcinogenesis
Source: Aging (Albany NY). 2021 Oct 25;13(20):23545–78. doi: 10.18632/aging.203651 (PMC8580351; doi:10.18632/aging.203651)
Supplement: Supplementary Tables [file aging-13-203651-s002.pdf]

SUPPLEMENTARY TABLES

Supplementary Table 1A. Male (M) parameter values of model-fits to SEER 2010–2013 age-specific cancer incidence rates for reproductive and non-reproductive cancer types, employing the multistage-senescence model (Equation 5).

Sex	Cancer type (TCGA)[0]	Model-fitted[1]			P values			Peak incidence rate (per 100,000 person-year)		Age of peak incidence (yr)		Cumulative probability over lifespan			Ratio of cum. prob., SEER/2-variable
		u_μ (yr^{-1})	*k*	*b* (yr^{-1})	*u*	*k*	*b*	SEER	Model-fitted[2]	SEER	Model-fitted[3]	SEER	Model-fitted[4]	2-Variable model fitted	
M	ACC	0.011	6.44	0.0107	3.60E-05	1.30E-07	5.50E-14	0.347	0.301	87.5	79.2	0.00012	9.50E-05	0.01	0.011
M	BLCA	0.04	9	0.0098	7.50E-12	3.60E-12	2.50E-15	344	380	92.5	91	0.1	0.1	0.017	6.1
M	COAD	0.029	6.9	0.0094	1.40E-10	3.00E-11	3.30E-13	228	230	92.5	90.9	0.08	0.078	0.023	3.5
M	COADREAD	0.027	6.19	0.0092	8.60E-10	2.10E-10	6.20E-12	274	279	92.5	91.2	0.1	0.11	0.026	3.9
M	ESCA	0.025	6.91	0.0101	5.00E-08	5.10E-09	2.90E-13	42.5	47.4	82.5	84.9	0.016	0.015	0.014	1.1
M	GBM	0.019	6.16	0.0103	2.00E-09	1.10E-10	2.70E-16	19.7	19.3	77.5	81.7	0.0067	0.0065	0.013	0.5
M	HNSC	0.019	5.07	0.01	1.00E-07	2.30E-08	1.00E-12	110	90.4	102.5	80.6	0.041	0.037	0.017	2.4
M	KICH	0.014	5.88	0.0106	1.90E-06	3.20E-08	4.10E-15	4.13	3.65	72.5	78.2	0.0013	0.0012	0.011	0.12
M	KIRC	0.017	5.13	0.0105	1.40E-06	2.70E-07	4.10E-15	41.9	37.9	72.5	76.9	0.014	0.014	0.013	1
M	KIRP	0.016	5.62	0.0106	2.00E-05	1.20E-06	1.30E-14	13.5	11.4	72.5	77.2	0.0039	0.004	0.011	0.34
M	LAML	0.03	8.37	0.0098	6.70E-12	5.30E-13	2.20E-16	42	45.5	87.5	89.5	0.014	0.013	0.016	0.85
M	LGG	9.20E-05	1.52	0.0085	8.80E-01	7.70E-01	8.40E-02	0.502	0.363	82.5	40.3	0.0003	0.0003	0.032	0.0094
M	LIHC	0.015	4.57	0.0102	3.50E-04	7.60E-05	2.30E-10	47.3	46.1	62.5	76.7	0.018	0.02	0.017	1.1
M	LUAD	0.035	7.81	0.0102	3.90E-09	2.40E-09	7.00E-15	202	206	82.5	85.6	0.059	0.058	0.012	4.8
M	LUSC	0.035	8.4	0.0102	5.40E-08	2.50E-08	1.60E-14	135	134	82.5	85.9	0.036	0.036	0.011	3.1
M	MESO	0.038	11.42	0.01	1.90E-10	1.10E-11	9.70E-16	11	12.7	92.5	91.5	0.0027	0.0027	0.014	0.19
M	PAAD	0.027	7.09	0.0101	1.00E-09	2.30E-10	1.00E-15	74.9	75.7	82.5	85.1	0.023	0.023	0.014	1.7
M	PCPG	7.00E-06	1.33	-0.0168	1.00E+00	1.00E+00	1.00E+00	0.407	NaN	92.5	-14.8	9.30E-05	NaN	NaN	NaN
M	READ	0.014	4.49	0.0089	1.60E-07	6.80E-09	1.60E-10	51	49.9	97.5	87.8	0.023	0.025	0.031	0.73
M	SARC	0.015	6.4	0.0071	4.70E-03	6.20E-05	1.70E-02	12.6	24.4	87.5	119	0.0042	0.012	0.13	0.031
M	SKCM	0.027	6.52	0.0093	3.90E-11	6.20E-12	1.40E-13	220	195	102.5	91	0.075	0.07	0.024	3.1
M	STAD	0.026	7.24	0.0096	3.00E-12	2.10E-13	1.30E-15	71.4	70.2	97.5	90.2	0.024	0.023	0.02	1.2
M	THCA	0.011	4.14	0.0103	1.20E-07	6.80E-09	1.30E-15	19.8	18.3	72.5	73.6	0.0087	0.0082	0.017	0.52
M	THYM	0.012	6.11	0.0107	7.10E-05	5.20E-07	1.00E-13	1	0.795	82.5	78.1	0.00028	0.00026	0.01	0.027
M	PRAD	0.035	5.11	0.0125	2.60E-06	9.90E-06	2.40E-11	746	642	72.5	79.5	0.22	0.21	0.0055	40
M	TGCT	0.0013	1.52	0.0237	8.00E-02	2.90E-02	2.70E-05	14.7	12.5	27.5	29.3	0.004	0.0036	0.0067	0.6

[0]Non-reproductive cancers: ACC adrenocortical carcinoma, BLCA bladder urothelial carcinoma, COAD colon adenocarcinoma, COADREAD both COAD and READ cases, ESCA esophageal carcinoma, GBM glioblastoma multiforme, HNSC head and neck squamous cell carcinoma, KICH kidney chromophobe, KIRC kidney renal clear cell carcinoma, KIRP kidney renal papillary cell carcinoma, LAML acute myeloid leukemia, LGG brain lower grade glioma, LIHC liver hepatocellular carcinoma, LUAD lung adenocarcinoma, LUSC lung squamous cell carcinoma, MESO mesothelioma, PAAD pancreatic adenocarcinoma, PCPG pheochromocytoma and paraganglioma, READ rectum adenocarcinoma, SARC sarcoma, SKCM skin cutaneous melanoma, STAD stomach adenocarcinoma, THYM thymoma, THCA thyroid carcinoma.

Male reproductive cancers: PRAD prostate adenocarcinoma, TGCT testicular germ cell tumors.

Other abbreviations: NaN Not a Number.

[1]Equation 5; [2]Equation 6; [3]Equation 6 substituted in Equation 5; [4]Equation 7.

The peak age-specific incidence rate, age of peak incidence rate, and cumulative probability of cancer over life span are computed up to maximum age 105, based on the SEER data alternatively model-fitted $ASR(t)$. The age-specific incidence rate is computed per 100,000 population for each five-year age group. LGG and PCPG are cancer types with P values for model-fitted u_μ or k larger than 0.1 and were omitted from further analysis.

Supplementary Table 1B. Female (F) parameter values of model-fits to SEER 2010–2013 age-specific cancer incidence rates for reproductive and non-reproductive cancer types, employing the multistage-senescence model (Equation 5).

Sex	Cancer type (TCGA)[0]	Model-fitted[1]			P values			Peak incidence rate (per 100,000 person-year)		Age of peak incidence (yr)		Cumulative probability over lifespan			Ratio of cum. prob., SEER/2-variable
		u_μ (yr^{-1})	*k*	*b* (yr^{-1})	*u*	*k*	*b*	SEER	Model-fitted[2]	SEER	Model-fitted[3]	SEER	Model-fitted[4]	2-Variable model fitted	
F	ACC	0.005	4.42	0.0105	1.60E-03	6.00E-06	3.70E-13	0.396	0.343	77.5	73.7	0.00015	0.00014	0.0077	0.02
F	BLCA	0.03	8.08	0.0097	1.10E-10	1.50E-11	1.70E-15	64.6	72.7	92.5	90.2	0.022	0.021	0.0071	3.1
F	COAD	0.03	7.23	0.0095	2.90E-09	9.40E-10	2.50E-13	193	188	87.5	90.2	0.062	0.061	0.0086	7.2
F	COADREAD	0.028	6.55	0.0093	6.50E-08	2.00E-08	2.70E-11	220	213	87.5	90.6	0.075	0.076	0.01	7.1
F	ESCA	0.02	7.09	0.0098	6.10E-10	1.70E-11	1.60E-15	11.7	12.1	87.5	88.1	0.0043	0.0039	0.0075	0.58
F	GBM	0.015	5.56	0.0101	5.20E-07	2.00E-08	9.50E-15	12.6	11.1	77.5	81.4	0.0042	0.0042	0.0075	0.56
F	HNSC	0.015	5.13	0.0092	9.70E-10	3.20E-11	4.70E-14	31.4	29.5	92.5	87.1	0.013	0.013	0.013	1
F	KICH	0.0083	4.74	0.0104	6.20E-04	8.00E-06	4.20E-13	2.21	1.86	77.5	75.6	0.00081	0.00075	0.0074	0.11
F	KIRC	0.013	4.63	0.0104	7.30E-05	9.00E-06	1.70E-15	23.2	18	72.5	75.6	0.0073	0.0075	0.0078	0.94
F	KIRP	0.011	5.22	0.0105	1.50E-04	3.00E-06	3.90E-14	3.56	2.98	77.5	77.3	0.0011	0.0011	0.0065	0.17
F	LAML	0.023	7.33	0.0097	2.90E-09	1.40E-10	1.50E-14	22	23.1	82.5	88.8	0.0082	0.0072	0.0075	1.1
F	LGG	0.00093	2.65	0.0101	5.60E-01	1.90E-01	6.90E-06	0.364	0.213	32.5	61.4	0.00022	0.00013	0.013	0.017
F	LIHC	0.018	6.06	0.0101	6.00E-08	3.50E-09	1.10E-15	17.3	16.2	82.5	82.4	0.0057	0.0056	0.0067	0.85
F	LUAD	0.029	6.73	0.0101	1.90E-07	1.30E-07	1.70E-14	162	144	77.5	84	0.047	0.046	0.006	7.8
F	LUSC	0.028	7.65	0.0102	6.80E-06	2.10E-06	8.60E-15	69	51.9	77.5	85.3	0.016	0.015	0.0051	3.1
F	MESO	0.018	7.85	0.0091	4.00E-05	2.80E-07	2.40E-08	2.29	2.15	102.5	96.3	0.00065	0.00068	0.012	0.052
F	PAAD	0.027	7.18	0.01	7.80E-09	1.70E-09	1.60E-15	60.7	59.5	82.5	86	0.019	0.018	0.0062	3
F	PCPG	0.004	4.45	0.0109	2.20E-01	1.60E-02	5.80E-09	0.182	0.111	72.5	71.1	6.10E-05	4.50E-05	0.0064	0.0094
F	READ	0.0043	2.82	0.0038	4.10E-01	1.50E-01	6.80E-01	28.3	49.3	82.5	170	0.013	0.076	0.2	0.063
F	SARC	0.0093	5.01	0.0077	5.60E-05	2.00E-07	1.80E-06	7.67	6.65	97.5	104	0.0023	0.0035	0.031	0.074
F	SKCM	0.012	4.08	0.0083	4.20E-06	2.20E-07	1.30E-08	59.4	58.8	82.5	91	0.029	0.033	0.022	1.3
F	STAD	0.022	6.98	0.0094	2.00E-08	9.10E-10	2.10E-12	34.4	33.1	102.5	91.2	0.012	0.011	0.0099	1.2
F	THCA	0.0053	2.36	0.0102	9.20E-09	7.10E-10	5.10E-19	38.3	36.6	52.5	56.3	0.022	0.022	0.013	1.7
F	THYM	0.0087	5.34	0.0107	1.20E-04	8.20E-07	8.20E-17	0.793	0.658	67.5	75.7	0.00025	0.00024	0.0056	0.045
F	BRCA	0.023	3.72	0.0115	1.20E-09	4.60E-09	9.00E-14	435	428	77.5	78.5	0.19	0.18	0.0065	30
F	CESC	0.0011	1.57	0.0105	1.50E-03	9.00E-05	2.50E-10	13.8	11.9	42.5	49.5	0.0071	0.0078	0.0094	0.76
F	OV	0.011	4.07	0.0121	1.20E-06	2.60E-08	8.30E-15	12.7	11.9	72.5	77.5	0.0045	0.0046	0.0048	0.96
F	UCEC	0.013	3.4	0.0121	8.60E-05	3.00E-05	1.00E-11	84	73.1	67.5	73.2	0.03	0.032	0.0061	5
F	UCS	0.01	5.59	0.0125	3.70E-03	3.00E-05	6.60E-11	0.711	0.519	82.5	80.6	0.00018	0.00016	0.0022	0.081

[0]Female reproductive cancers: BRCA breast invasive carcinoma, CESC cervical squamous cell carcinoma and endocervical adenocarcinoma, OV ovarian serous cystadenocarcinoma, UCEC uterine corpus endometrial carcinoma, UCS uterine carcinosarcoma.

Abbreviations for non-reproductive cancer types and footnotes 1-4 are explained in Supplementary Table 1A. LGG, PCPG and READ are cancer types with *P* values for model-fitted u_μ or *k* larger than 0.1 and were omitted from further analysis.

Supplementary Table 1C. Both sexes pooled (M&F) parameter values of model-fits to SEER 2010–2013 age-specific cancer incidence rates for non-reproductive cancer types only, employing the multistage-senescence model (Equation 5).

Sex	Cancer type (TCGA)[0]	Model-fitted[1]			P values			Peak incidence rate (per 100,000 person-year)		Age of peak incidence (yr)		Cumulative probability over lifespan			Ratio of cum. prob., SEER/2-variable
		u_μ (yr^{-1})	*k*	*b* (yr^{-1})	*u*	*k*	*b*	SEER	Model-fitted[2]	SEER	Model-fitted[3]	SEER	Model-fitted[4]	2-Variable model fitted	
M&F	ACC	0.0067	5.1	0.0105	9.30E-06	1.80E-08	1.70E-15	0.329	0.319	77.5	76.6	0.00014	0.00012	0.0064	0.021
M&F	BLCA	0.036	8.47	0.0098	1.30E-10	5.20E-11	1.80E-15	161	183	87.5	89.6	0.052	0.05	0.013	4.1
M&F	COAD	0.03	7.03	0.0095	4.20E-10	1.20E-10	1.00E-13	203	203	87.5	90.1	0.068	0.067	0.014	5
M&F	COADREAD	0.027	6.32	0.0093	4.80E-09	1.50E-09	4.30E-12	238	235	87.5	90.2	0.084	0.086	0.014	6
M&F	ESCA	0.022	6.64	0.01	4.50E-08	3.10E-09	3.50E-14	24.1	26	82.5	84.7	0.009	0.0085	0.0092	0.98
M&F	GBM	0.016	5.65	0.0101	1.50E-07	7.20E-09	4.30E-15	15.7	14.3	77.5	81.6	0.0052	0.0053	0.0082	0.63
M&F	HNSC	0.017	4.87	0.0098	9.20E-08	1.20E-08	2.80E-13	52.6	54.3	77.5	80.9	0.024	0.023	0.0088	2.7
M&F	KICH	0.01	5.17	0.0105	4.50E-05	6.10E-07	1.90E-14	3.05	2.58	72.5	77	0.001	0.00098	0.0065	0.16
M&F	KIRC	0.014	4.69	0.0104	2.40E-05	4.10E-06	2.90E-15	31.7	26.1	72.5	75.8	0.01	0.011	0.0066	1.5
M&F	KIRP	0.012	5.05	0.0105	2.40E-04	1.00E-05	6.00E-14	8.04	6.33	72.5	76.4	0.0024	0.0024	0.0064	0.37
M&F	LAML	0.026	7.77	0.0098	2.40E-10	1.70E-11	1.30E-15	29.6	31.4	82.5	88.8	0.01	0.0093	0.012	0.85
M&F	LGG	0.00064	2.34	0.0099	5.00E-01	1.40E-01	2.10E-06	0.404	0.279	32.5	57.9	0.00026	0.00018	0.0026	0.1
M&F	LIHC	0.014	4.69	0.0102	6.00E-05	7.80E-06	3.00E-12	28.4	28.7	77.5	77.5	0.011	0.012	0.0073	1.5
M&F	LUAD	0.03	7.07	0.0101	7.10E-08	4.80E-08	2.10E-14	177	165	77.5	85	0.051	0.051	0.0091	5.7
M&F	LUSC	0.03	7.69	0.0101	1.60E-06	6.20E-07	3.30E-14	95.8	81.4	77.5	85.8	0.024	0.023	0.0092	2.6
M&F	MESO	0.03	10.2	0.0099	8.60E-10	3.10E-11	4.50E-16	5.09	5.69	87.5	90.8	0.0014	0.0013	0.014	0.1
M&F	PAAD	0.026	7.02	0.01	5.40E-09	1.20E-09	2.00E-15	66.5	65.7	82.5	85.5	0.02	0.021	0.0095	2.2
M&F	PCPG	0.0032	4.11	0.0103	1.00E-01	2.00E-03	1.80E-08	0.188	0.133	92.5	73.4	7.30E-05	6.00E-05	0.0065	0.011
M&F	READ	0.011	4.12	0.0087	1.50E-05	5.80E-07	8.10E-09	37	35.8	82.5	87.5	0.016	0.019	0.013	1.2
M&F	SARC	0.014	6.06	0.0084	2.10E-06	1.30E-08	1.80E-08	8.74	8.96	97.5	98.9	0.0029	0.0037	0.025	0.12
M&F	SKCM	0.021	5.67	0.0094	1.50E-10	2.20E-11	3.00E-14	107	107	82.5	87.9	0.045	0.043	0.013	3.6
M&F	STAD	0.024	7.02	0.0096	5.40E-11	3.50E-12	3.00E-15	43.3	46.5	87.5	89.5	0.016	0.015	0.013	1.3
M&F	THCA	0.0059	2.69	0.0101	1.10E-04	2.10E-05	2.40E-13	28.6	26.4	67.5	62.4	0.016	0.016	0.0042	3.7
M&F	THYM	0.0094	5.54	0.0106	4.20E-05	2.60E-07	6.80E-16	0.747	0.705	67.5	77	0.00026	0.00025	0.0061	0.043
**M**	**Mean**	0.0231	6.8	0.0100				42.3	45.8	82.5	85.0	0.015	0.015	0.014	1.06
**M**	**SD**	0.0092	1.7	0.0008				95.9	99.6	10.7	9.8	0.030	0.029	0.027	1.73
**F**	**Mean**	0.0174	5.9	0.0098				22.6	20.6	82.5	85.6	0.0078	0.0073	0.0075	0.97
**F**	**SD**	0.0084	1.5	0.0008				52.1	49.4	11.8	10.0	0.016	0.016	0.062	2.22
μ_M, μ_F[5]	***t* test, *P***	0.000056	0.0033	0.034*				0.014†	0.012†	0.45	0.32	0.012†	0.0094†	0.035*	0.73

[5]The mean, SD and paired *t*-test *P* values in each column are for the 20 non-reproductive, paired cancer types for males and females, for the parameter in the corresponding column.

†Values are no longer significant to 5% level when Holm's method for multiple-testing correction is applied for the paired *t* tests in the table

*Values are no longer significant to 10% level when Holm's method for multiple-testing correction is applied for the paired *t* tests in the table.

Male (M) and female (F) parameter values are compared by paired *t* test for 20 paired cancer types in the last row of the table.

Abbreviations for non-reproductive cancer types (footnote 0) and footnotes 1-4 are explained in Supplementary Table 1A. LGG and PCPG are cancer types with *P* values for model-fitted u_μ or *k* larger than 0.1 and were omitted from further analysis.

Supplementary Table 2A. Male (M) parameter values of model-fits to SEER 2000–2003 age-specific cancer incidence rates for reproductive and non-reproductive cancer types, employing the multistage-senescence model (Equation 5).

Sex	Cancer type (TCGA)[0]	Model-fitted[1]			*P* values			Peak incidence rate (per 100,000 person-year)		Age of peak incidence (yr)		Cumulative probability over lifespan			Ratio of cum. prob., SEER/2-variable
		u_μ (yr^{-1})	k	b (yr^{-1})	u	k	b	SEER	Model-fitted[2]	SEER	Model-fitted[3]	SEER	Model-fitted[4]	2-Variable model fitted	
M	ACC	0.0077	5.43	0.0106	9.20E-02	3.90E-03	3.00E-07	0.456	0.289	72.5	76.6	0.00014	0.0001	0.01	0.014
M	BLCA	0.037	8.2	0.0096	7.80E-11	4.20E-11	5.40E-14	322	363	87.5	91.2	0.1	0.1	0.023	4.3
M	COAD	0.035	7.63	0.0096	7.20E-12	3.70E-12	9.30E-15	346	374	87.5	91	0.11	0.12	0.023	4.8
M	COADREAD	0.034	7.22	0.0096	3.60E-12	2.40E-12	5.60E-15	420	442	87.5	90.1	0.14	0.14	0.022	6.2
M	ESCA	0.024	6.59	0.0101	1.80E-09	1.90E-10	8.60E-15	50.2	48	102.5	84.2	0.018	0.016	0.014	1.3
M	GBM	0.019	6.08	0.0102	2.50E-08	1.30E-09	9.80E-15	18.9	18.7	77.5	81.5	0.0066	0.0064	0.013	0.52
M	HNSC	0.019	5.04	0.0099	1.50E-07	3.30E-08	2.60E-12	86.9	92.2	72.5	80.7	0.039	0.038	0.014	2.7
M	KICH	0.014	6.31	0.0106	1.30E-07	1.10E-09	5.10E-16	1.93	1.72	77.5	79.5	0.00056	0.00056	0.01	0.054
M	KIRC	0.015	5.07	0.0104	3.10E-07	2.80E-08	8.00E-16	21.9	20.3	72.5	77.2	0.0073	0.0078	0.012	0.63
M	KIRP	0.015	5.87	0.0107	4.30E-07	1.10E-08	1.00E-16	5.17	5.02	72.5	77.6	0.0016	0.0017	0.0099	0.16
M	LAML	0.028	8.09	0.0099	5.00E-12	3.90E-13	1.10E-16	38	39.1	87.5	88.6	0.012	0.011	0.018	0.67
M	LGG	0.00026	1.71	0.0073	8.10E-01	6.40E-01	2.10E-01	1.07	0.864	77.5	56.8	0.00072	0.00082	0.0044	0.16
M	LIHC	0.015	4.97	0.01	4.50E-08	3.10E-09	5.40E-14	32.2	30.6	77.5	79.9	0.012	0.012	0.014	0.85
M	LUAD	0.031	7.17	0.0102	6.40E-08	4.00E-08	1.20E-13	168	172	77.5	84.1	0.052	0.052	0.013	3.9
M	LUSC	0.033	7.84	0.0103	5.60E-07	3.00E-07	3.80E-13	139	136	77.5	85	0.038	0.038	0.013	2.8
M	MESO	0.033	10.02	0.0102	3.70E-08	2.50E-09	9.10E-14	12.6	14.8	82.5	88.6	0.004	0.0034	0.017	0.23
M	PAAD	0.026	6.97	0.0102	5.50E-09	1.00E-09	4.00E-15	55.4	57.5	82.5	83.7	0.018	0.018	0.013	1.4
M	PCPG	2.60E-10	0.47	0.0092	9.90E-01	8.90E-01	3.70E-01	0.326	NaN	92.5	-124	7.50E-05	0.00023	NaN	NaN
M	READ	0.023	6.2	0.0098	5.60E-12	7.80E-13	1.60E-16	73.8	75.2	87.5	85.2	0.026	0.026	0.016	1.6
M	SARC	0.017	6.82	0.0088	2.90E-04	3.10E-06	4.50E-06	7.67	7.57	97.5	97.3	0.0022	0.0028	0.038	0.058
M	SKCM	0.02	5.39	0.009	1.60E-11	1.50E-12	8.10E-14	123	123	92.5	90.3	0.05	0.053	0.025	2
M	STAD	0.028	7.51	0.0096	2.20E-11	2.00E-12	2.20E-14	100	90.2	102.5	90.8	0.032	0.028	0.023	1.4
M	THCA	0.009	3.97	0.0103	2.10E-07	5.60E-09	3.10E-15	11.8	11.1	67.5	72.8	0.0053	0.0052	0.012	0.44
M	THYM	0.0093	5.52	0.0104	2.10E-05	7.60E-08	6.10E-13	0.884	0.756	82.5	78.6	0.00029	0.00027	0.011	0.026
M	PRAD	0.038	5.44	0.012	3.00E-07	1.50E-06	1.10E-10	1020	1010	72.5	83.1	0.33	0.32	0.0053	61
M	TGCT	0.0019	1.69	0.0238	1.00E-02	1.90E-03	3.10E-06	12.6	11.6	32.5	32.2	0.0037	0.0034	0.00054	6.8

[0]Non-reproductive cancers: ACC adrenocortical carcinoma, BLCA bladder urothelial carcinoma, COAD colon adenocarcinoma, COADREAD both COAD and READ cases, ESCA esophageal carcinoma, GBM glioblastoma multiforme, HNSC head and neck squamous cell carcinoma, KICH kidney chromophobe, KIRC kidney renal clear cell carcinoma, KIRP kidney renal papillary cell carcinoma, LAML acute myeloid leukemia, LGG brain lower grade glioma, LIHC liver hepatocellular carcinoma, LUAD lung adenocarcinoma, LUSC lung squamous cell carcinoma, MESO mesothelioma, PAAD pancreatic adenocarcinoma, PCPG pheochromocytoma and paraganglioma, READ rectum adenocarcinoma, SARC sarcoma, SKCM skin cutaneous melanoma, STAD stomach adenocarcinoma, THYM thymoma, THCA thyroid carcinoma.
Male reproductive cancers: PRAD prostate adenocarcinoma, TGCT testicular germ cell tumors.
Other abbreviations: NaN Not a Number.
[1]Equation 5; [2]Equation 6; [3]Equation 6 substituted in Equation 5; [4]Equation 7.
The peak age-specific incidence rate, age of peak incidence rate, and cumulative probability of cancer over life span are computed up to maximum age 105, based on the SEER data alternatively model-fitted $ASR(t)$. The age-specific incidence rate is computed per 100,000 population for each five-year age group. LGG and PCPG are cancer types with *P* values for model-fitted u_μ or k larger than 0.1 and were omitted from further analysis.

Supplementary Table 2B. Female (F) parameter values of model-fits to SEER 2000–2003 age-specific cancer incidence rates for reproductive and non-reproductive cancer types, employing the multistage-senescence model (Equation 5).

Sex	Cancer type (TCGA)[0]	Model-fitted[1]			*P* values			Peak incidence rate (per 100,000 person-year)		Age of peak incidence (yr)		Cumulative probability over lifespan			Ratio of cum. prob., SEER/2-variable
		u_μ (yr^{-1})	*k*	*b* (yr^{-1})	*u*	*k*	*b*	SEER	Model -fitted[2]	SEER	Model-fitted[3]	SEER	Model-fitted[4]	2-Variable model fitted	
F	ACC	0.0019	3.2	0.0105	2.30E-01	1.70E-02	8.40E-09	0.383	0.24	62.5	65.4	0.00013	0.00012	0.011	0.011
F	BLCA	0.028	7.67	0.0096	1.40E-10	2.10E-11	5.20E-15	70.5	76	87.5	90.3	0.024	0.023	0.0079	3
F	COAD	0.033	7.59	0.0095	4.00E-12	2.30E-12	5.80E-16	288	291	87.5	91.4	0.088	0.091	0.0088	9.9
F	COADREAD	0.032	7.25	0.0094	2.30E-12	1.50E-12	5.90E-16	334	335	87.5	91.2	0.1	0.11	0.0094	11
F	ESCA	0.022	7.32	0.0097	1.80E-07	6.70E-09	2.10E-12	14.7	15.5	87.5	88.8	0.0054	0.0048	0.0077	0.7
F	GBM	0.015	5.61	0.0102	1.10E-07	4.50E-09	6.00E-16	11.9	10.5	77.5	80.8	0.0038	0.0039	0.0074	0.51
F	HNSC	0.018	5.66	0.0097	4.20E-08	2.60E-09	2.20E-13	30.6	31.6	77.5	84.6	0.013	0.012	0.0093	1.4
F	KICH	0.0098	5.57	0.0105	4.20E-04	4.30E-06	1.20E-13	0.996	0.863	82.5	78	0.00033	0.00031	0.0061	0.054
F	KIRC	0.011	4.54	0.0104	6.10E-05	3.60E-06	4.10E-15	11.6	9.78	72.5	74.7	0.0038	0.0041	0.0081	0.46
F	KIRP	0.0098	5.44	0.0105	5.70E-05	4.90E-07	1.80E-14	1.45	1.16	77.5	77.8	0.00043	0.00042	0.0064	0.067
F	LAML	0.02	6.76	0.0096	1.70E-11	7.00E-13	9.30E-17	20.4	19.5	87.5	88.4	0.007	0.0066	0.0086	0.8
F	LGG	0.00052	2.06	0.0089	4.30E-01	1.00E-01	1.50E-05	2.58	0.59	102.5	57.8	0.0007	0.00044	0.021	0.034
F	LIHC	0.019	6.63	0.0102	3.90E-07	2.30E-08	2.60E-15	11.2	11.5	77.5	82.9	0.0039	0.0037	0.0058	0.67
F	LUAD	0.025	6.06	0.0102	1.20E-06	7.20E-07	8.60E-14	117	104	77.5	81.8	0.036	0.036	0.0066	5.5
F	LUSC	0.026	7.15	0.0103	1.80E-05	5.20E-06	9.40E-14	54.1	44.8	77.5	83.6	0.014	0.014	0.0052	2.8
F	MESO	0.02	7.99	0.0102	4.90E-07	1.10E-08	1.80E-16	2.38	1.99	82.5	85.4	0.00055	0.00055	0.0047	0.12
F	PAAD	0.023	6.68	0.0099	1.60E-07	3.40E-08	5.80E-15	43.6	43.2	77.5	85.6	0.014	0.014	0.0071	1.9
F	PCPG	0.0015	3.24	0.0108	4.40E-01	7.90E-02	2.40E-08	0.141	0.0863	72.5	64	5.00E-05	4.30E-05	0.01	0.0049
F	READ	0.019	5.85	0.0093	1.90E-12	1.10E-13	1.80E-16	46.6	45.1	87.5	89	0.017	0.018	0.012	1.5
F	SARC	0.0057	4.25	0.0054	1.50E-01	7.00E-03	3.10E-01	5.54	8.09	97.5	143	0.0016	0.0069	0.15	0.011
F	SKCM	0.0085	3.43	0.0078	5.50E-06	1.80E-07	2.70E-08	42.1	42.3	82.5	91	0.024	0.028	0.03	0.79
F	STAD	0.026	7.62	0.0094	9.30E-11	6.20E-12	1.20E-14	44.7	44.6	87.5	92.5	0.014	0.014	0.0096	1.4
F	THCA	0.0033	2.15	0.0098	1.40E-10	2.00E-12	2.90E-20	20.7	20.3	52.5	54.7	0.012	0.014	0.017	0.73
F	THYM	0.007	5	0.0104	8.40E-03	1.00E-04	4.00E-11	0.724	0.502	72.5	76.6	0.00021	0.0002	0.0073	0.029
F	BRCA	0.022	3.43	0.0112	2.30E-11	1.10E-10	6.30E-15	454	440	77.5	78	0.2	0.2	0.0084	24
F	CESC	0.0013	1.6	0.0098	6.50E-05	1.70E-06	9.10E-11	15.5	14.5	42.5	53.2	0.0095	0.01	0.015	0.64
F	OV	0.008	4	0.0122	5.30E-06	3.90E-08	1.60E-14	4.34	4.08	77.5	76.6	0.0016	0.0016	0.0051	0.31
F	UCEC	0.014	3.62	0.0119	9.50E-07	2.20E-07	5.50E-13	74.4	74.3	67.5	76	0.031	0.032	0.0065	4.8
F	UCS	0.0067	4.96	0.0121	8.70E-04	1.50E-06	7.70E-11	0.426	0.237	97.5	81.2	9.80E-05	8.00E-05	0.0036	0.027

[0]Female reproductive cancers: BRCA breast invasive carcinoma, CESC cervical squamous cell carcinoma and endocervical adenocarcinoma, OV ovarian serous cystadenocarcinoma, UCEC uterine corpus endometrial carcinoma, UCS uterine carcinosarcoma.

Abbreviations for non-reproductive cancer types and footnotes 1-4 are explained in Supplementary Table 2A. ACC, LGG, PCPG and SARC are cancer types with *P* values for model-fitted u_μ or *k* larger than 0.1 and were omitted from further analysis.

Supplementary Table 2C. Both sexes pooled (M&F) parameter values of model-fits to SEER 2000–2003 age-specific cancer incidence rates for non-reproductive cancer types only, employing the multistage-senescence model (Equation 5).

Sex	Cancer type (TCGA)[0]	Model-fitted[1]			*P* values			Peak incidence rate (per 100,000 person-year)		Age of peak incidence (yr)		Cumulative probability over lifespan			Ratio of cum. prob., SEER/2-variable
		u_μ (yr^{-1})	*k*	*b* (yr^{-1})	*u*	*k*	*b*	SEER	Model-fitted[2]	SEER	Model-fitted[3]	SEER	Model-fitted	2-Variable model fitted[4]	
M&F	ACC	0.0036	4.01	0.0104	1.20E-01	4.20E-03	2.70E-08	0.343	0.246	62.5	72	0.00013	0.00011	0.0047	0.027
M&F	BLCA	0.032	7.64	0.0097	1.60E-09	6.60E-10	5.70E-14	153	171	82.5	89.5	0.051	0.052	0.015	3.3
M&F	COAD	0.033	7.44	0.0095	1.10E-11	6.40E-12	3.70E-15	307	321	87.5	91.5	0.096	0.1	0.018	5.4
M&F	COADREAD	0.032	7.06	0.0094	5.60E-12	3.90E-12	2.90E-15	363	372	87.5	90.9	0.12	0.12	0.016	7.1
M&F	ESCA	0.021	6.37	0.01	5.20E-08	3.60E-09	9.10E-14	24.5	27.2	82.5	84.5	0.01	0.0092	0.0099	1
M&F	GBM	0.016	5.62	0.0101	1.50E-07	7.50E-09	3.30E-15	14.8	13.6	77.5	81.1	0.0049	0.005	0.0078	0.62
M&F	HNSC	0.017	4.92	0.0099	2.50E-07	3.80E-08	5.50E-13	53.4	55.1	72.5	80.4	0.023	0.023	0.0076	3.1
M&F	KICH	0.011	5.66	0.0105	1.60E-06	1.10E-08	2.70E-16	1.36	1.19	77.5	78.5	0.00043	0.00042	0.0065	0.066
M&F	KIRC	0.012	4.58	0.0104	1.80E-05	1.40E-06	2.70E-15	16.2	13.8	72.5	75.5	0.0053	0.0058	0.0057	0.93
M&F	KIRP	0.011	5.18	0.0105	3.00E-05	4.90E-07	2.30E-15	2.94	2.65	72.5	76.9	0.00094	0.001	0.006	0.16
M&F	LAML	0.024	7.33	0.0098	1.10E-11	6.90E-13	4.20E-17	26.2	26.4	87.5	88.1	0.0088	0.0082	0.013	0.66
M&F	LGG	0.00099	2.37	0.0094	2.80E-01	3.70E-02	4.70E-07	2.05	0.73	102.5	61.3	0.00074	0.00049	0.00051	1.5
M&F	LIHC	0.015	5.17	0.0102	6.00E-08	3.90E-09	1.10E-15	19.9	18.9	77.5	79.4	0.0072	0.0074	0.0071	1
M&F	LUAD	0.027	6.39	0.0102	7.20E-07	4.50E-07	1.90E-13	138	127	77.5	83.1	0.042	0.042	0.0089	4.7
M&F	LUSC	0.028	7.19	0.0102	6.90E-06	2.90E-06	1.90E-13	89.3	76.3	77.5	84.1	0.024	0.023	0.0095	2.5
M&F	MESO	0.026	8.92	0.0102	1.00E-07	4.60E-09	4.20E-15	6.28	6.32	82.5	87.2	0.0017	0.0016	0.013	0.13
M&F	PAAD	0.024	6.66	0.01	8.40E-08	1.60E-08	1.50E-14	48.3	48.4	77.5	84.7	0.015	0.016	0.01	1.5
M&F	PCPG	9.70E-08	0.77	0.005	9.90E-01	9.50E-01	8.60E-01	0.132	NaN	67.5	-59.4	5.70E-05	0.00014	NaN	NaN
M&F	READ	0.021	5.9	0.0096	2.90E-12	2.80E-13	6.90E-17	55.5	56	87.5	86.2	0.021	0.021	0.011	1.9
M&F	SARC	0.012	5.87	0.0086	2.00E-04	1.40E-06	7.20E-07	5.99	5.14	97.5	96.4	0.0018	0.0022	0.021	0.085
M&F	SKCM	0.016	4.73	0.0092	8.40E-11	7.70E-12	1.30E-14	70.8	69.6	82.5	85.6	0.033	0.032	0.01	3.2
M&F	STAD	0.026	7.3	0.0095	1.70E-12	1.50E-13	2.10E-16	56.4	59.6	87.5	90.7	0.02	0.019	0.016	1.2
M&F	THCA	0.0046	2.67	0.0097	4.80E-07	2.20E-08	2.20E-15	15.6	15.1	67.5	64.2	0.0092	0.0093	0.0017	5.2
M&F	THYM	0.0079	5.18	0.0104	5.70E-05	2.30E-07	5.60E-14	0.729	0.612	72.5	77.3	0.00025	0.00023	0.0062	0.04

[0]Abbreviations for non-reproductive cancer types and footnotes 1-4 are explained in Supplementary Table 2A. ACC, LGG, and PCPG are cancer types with *P* values for model-fitted u_μ or *k* larger than 0.1 and were omitted from further analysis.

Supplementary Table 3. Correspondence of cancer types in TCGA and SEER, from Wang et al. (2018).

TCGA code	TCGA cancer type	SEER ICD-O-3 site (histology) code
ACC	Adrenocortical carcinoma	C74.0-C74.9 (8370/3)
BLCA	Bladder urothelial carcinoma	C67.0-C67.9 (8050, 8120, 8130, 8131)
BRCA	Breast invasive carcinoma	C50.0-C50.9 (801-857)
CESC	Cervical squamous cell carcinoma and endocervical adenocarcinoma	C53.0-C53.9 (807, 814-857)
COAD	Colon adenocarcinoma	C18.0-C18.9 (814-857)
ESCA	Esophageal carcinoma	C15.0-C15.9 (801-857)
GBM	Glioblastoma multiforme	(9440/3)
HNSC	Head and neck squamous cell carcinoma	C00.0-C15.0, C15.3, C30.0, C31.0-C33.9, C41.0, C41.1, C47.0, C49.0, C73.9, C75.4 C77.0 (807)
KICH	Kidney chromophobe	C64.9, C65.9 (8317, 8270)
KIRC	Kidney renal clear cell carcinoma	C64.9, C65.9 (8310/3)
KIRP	Kidney renal papillary cell carcinoma	C64.9, C65.9 (8260
LAML	Acute myeloid leukemia	ICCC site recode ICD-O-3/WHO 2008=I(b) Acute myeloid leukemias
LGG	Brain lower grade glioma	C71.0-C71.9 (9380-9384, 9391-9460, Grade I and II)
LIHC	Liver hepatocellular carcinoma	C22.0 (817-818)
LUAD	Lung adenocarcinoma	C34.0-C34.9 (814-857)
LUSC	Lung squamous cell carcinoma	C34.0-C34.9 (807)
MESO	Mesothelioma	(905)
OV	Ovarian serous cystadenocarcinoma	C56.9 (8441.3)
PAAD	Pancreatic adenocarcinoma	C25.0-C25.9 (814-857)
PCPG	Pheochromocytoma and paraganglioma	(868, 869, 870)
PRAD	Prostate adenocarcinoma	C61.9 (814-857)
READ	Rectum adenocarcinoma	C61.9 (814-857)
SARC	Sarcoma	(880)
SKCM	Skin cutaneous melanoma	C44.0-C44.9 (8720-8790)
STAD	Stomach adenocarcinoma	C16.0-C16.9 (814-857)
TGCT	Testicular germ cell tumors	C62.0-C62.9 (9060-9100)
THCA	Thyroid carcinoma	C73.9 (801-857)
THYM	Thymoma	C37.9 (8580-8585)
UCEC	Uterine corpus endometrial carcinoma	C54.0-C54.9 (8140, 8380, 8382, 8480, 8482, 8560, 8570, 8310, 8441, 8460, 8260)
UCS	Uterine carcinosarcoma	C55.9 (8980-8981)

Red and blue text for female and male reproductive cancers, respectively.

Supplementary Table 4A. Male (M) parameter values of fits to SEER 2010-2013 age-specific cancer incidence rates employing the multistage-senescence model of Equation 4 (Pompei and Wilson, 2001; Harding et al. 2008).

Sex	Cancer type	Model -fitted			Peak incidence rate (per 100,000 person-year)		Age of peak incidence (yr)		Cumulative probability over lifespan	
		a	*k*	*b* (yr^{-1})	SEER	Model-fitted	SEER	Model-fitted	SEER	Model-fitted
M	All Major	1.48E-12	6.75	0.0099	2682.3	2943.1	92.5	86.1	1.103	0.965
M	All Major Non Sex	6.07E-13	6.86	0.0096	2243.7	2326.6	92.5	89.2	0.837	0.778
M	All Sites	1.50E-12	6.76	0.0098	3016.5	3220.0	92.5	86.7	1.205	1.062
M	Brain and Other Nervous System	2.70E-12	5.54	0.0097	40.1	27.7	102.5	84.4	0.014	0.011
M	Colon and Rectum	6.42E-12	5.77	0.0083	354.6	372.2	92.5	99.4	0.125	0.164
M	Esophagus	1.47E-14	6.88	0.0100	44.3	48.6	92.5	85.5	0.017	0.016
M	[1] Hodgkin Lymphoma	2.45E-07	2.20	0.0000	5.4	NA	87.5	Inf	0.003	Inf
M	Kidney and Renal Pelvis	2.82E-12	5.85	0.0100	92.1	99.5	77.5	83.0	0.041	0.036
M	Larynx	1.35E-13	6.30	0.0102	29.0	31.5	77.5	82.7	0.012	0.011
M	Leukemia	2.22E-15	7.44	0.0094	143.4	140.1	92.5	92.5	0.047	0.045
M	Liver and Intrahepatic Bile Duct	1.91E-10	4.77	0.0099	56.7	58.5	82.5	79.7	0.024	0.025
M	Lung and Bronchus	4.60E-16	8.22	0.0101	512.1	563.0	82.5	87.2	0.172	0.155
M	Melanoma of the Skin	2.83E-13	6.42	0.0091	230.4	206.8	102.5	93.2	0.077	0.077
M	Myeloma	6.37E-16	7.63	0.0099	59.0	64.6	82.5	87.7	0.019	0.019
M	Non-Hodgkin Lymphoma	3.34E-14	6.90	0.0095	160.5	159.3	92.5	89.8	0.055	0.053
M	Oral Cavity and Pharynx	8.44E-10	4.43	0.0095	100.2	68.6	102.5	81.4	0.035	0.032
M	Pancreas	3.33E-15	7.36	0.0096	116.2	117.6	92.5	89.6	0.039	0.037
M	Stomach	6.10E-15	7.11	0.0094	84.6	80.8	97.5	91.5	0.027	0.027
M	Thyroid	1.58E-09	4.02	0.0103	18.2	17.0	72.5	73.2	0.008	0.008
M	Urinary Bladder	6.02E-18	9.05	0.0097	396.7	416.6	92.5	91.8	0.122	0.110
M	Prostate	6.57E-10	5.27	0.0124	749.7	691.7	72.5	80.1	0.261	0.218
M	Testis	5.23E-05	1.49	0.0236	15.1	12.8	27.5	29.0	0.004	0.004

NA Not Available; Inf Infinity.

[1]Hodgkin's lymphoma has a bimodal, rather than a unimodal distribution, thus does not well fit the multistage-senescence model.

The peak age-specific incidence rate, age of peak incidence, and cumulative probability over life span are computed up to maximum age 105, based on the SEER data alternatively model-fitted *ASR*(*t*).

Supplementary Table 4B. Female (F) parameter values of fits to SEER 2010-2013 age-specific cancer incidence rates employing the multistage-senescence model of Equation 4 (Pompei and Wilson, 2001; Harding et al. 2008).

Sex	Cancer type	Model -fitted			Peak incidence rate (per 100,000 person-year)		Age of peak incidence (yr)		Cumulative probability over lifespan	
		a	*k*	*b* (yr^{-1})	SEER	Model-fitted	SEER	Model-fitted	SEER	Model-fitted
F	All Major	2.51E-10	5.43	0.0095	1684.0	1740.1	82.5	85.9	0.739	0.702
F	All Major Non Sex	2.71E-12	6.37	0.0095	1161.7	1224.0	82.5	88.6	0.467	0.437
F	All Sites	2.28E-10	5.47	0.0094	1860.1	1936.8	82.5	86.9	0.821	0.785
F	Brain and Other Nervous System	8.51E-12	5.17	0.0095	17.8	17.9	77.5	84.8	0.009	0.007
F	Colon and Rectum	1.80E-12	6.00	0.0083	281.6	304.7	92.5	100.8	0.094	0.131
F	Esophagus	1.16E-15	7.10	0.0096	16.1	13.2	102.5	89.7	0.005	0.004
F	[1]Hodgkin Lymphoma	1.92E-11	4.61	0.0096	4.4	3.3	22.5	81.7	0.002	0.001
F	Kidney and Renal Pelvis	3.74E-12	5.61	0.0098	46.6	48.0	77.5	83.8	0.021	0.018
F	Larynx	2.69E-11	4.65	0.0102	5.1	4.5	77.5	77.0	0.002	0.002
F	Leukemia	9.14E-15	6.97	0.0092	80.3	74.3	102.5	93.3	0.027	0.026
F	Liver and Intrahepatic Bile Duct	9.93E-14	6.29	0.0097	27.9	28.0	82.5	86.5	0.010	0.010
F	Lung and Bronchus	1.15E-14	7.37	0.0100	335.2	342.6	82.5	86.4	0.110	0.104
F	Melanoma of the Skin	3.14E-09	3.98	0.0080	60.7	61.0	87.5	94.1	0.029	0.036
F	Myeloma	1.03E-14	6.87	0.0099	34.3	34.7	82.5	86.1	0.012	0.011
F	Non-Hodgkin Lymphoma	4.61E-14	6.74	0.0097	96.3	98.4	82.5	87.7	0.035	0.033
F	Oral Cavity and Pharynx	7.32E-11	4.70	0.0082	37.1	33.1	92.5	95.8	0.014	0.017
F	Pancreas	4.26E-16	7.77	0.0095	104.1	107.5	92.5	92.0	0.034	0.033
F	Stomach	9.39E-15	6.82	0.0091	48.2	42.2	102.5	94.1	0.015	0.015
F	Thyroid	4.22E-06	2.31	0.0103	37.7	35.6	52.5	55.3	0.021	0.022
F	Urinary Bladder	8.90E-17	8.08	0.0095	80.2	85.3	92.5	91.8	0.027	0.025
F	Breast	2.33E-07	3.67	0.0113	436.5	436.2	77.5	79.6	0.205	0.194
F	Cervix Uteri	3.10E-05	1.50	0.0093	14.4	12.5	42.5	50.9	0.008	0.009
F	Corpus Uteri	1.13E-07	3.50	0.0120	100.8	89.2	67.5	74.4	0.037	0.038
F	Ovary	1.48E-08	3.74	0.0102	48.2	47.8	87.5	87.0	0.022	0.023

[1]Hodgkin's lymphoma has a bimodal, rather than a unimodal distribution, thus does not well fit the multistage-senescence model.

Supplementary Table 4C. Male and female pooled (M&F) parameter values of fits to SEER 2010-2013 age-specific cancer incidence rates employing the multistage-senescence model of Equation 4 (Pompei and Wilson, 2001; Harding et al. 2008).

Sex	Cancer type	Model -fitted			Peak incidence rate (per 100,000 person-year)		Age of peak incidence (yr)		Cumulative probability over lifespan	
		a	*k*	*b* (yr^{-1})	SEER	Model-fitted	SEER	Model-fitted	SEER	Model-fitted
M&F	All Major	2.06E-11	6.08	0.0098	2052.5	2219.4	82.5	85.6	0.865	0.800
M&F	All Major Non Sex	1.51E-12	6.58	0.0096	1544.7	1633.9	82.5	87.9	0.598	0.561
M&F	All Sites	1.89E-11	6.11	0.0097	2253.5	2439.4	82.5	86.2	0.953	0.881
M&F	Brain and Other Nervous System	5.92E-12	5.31	0.0097	21.8	21.9	77.5	84.1	0.011	0.009
M&F	Colon and Rectum	3.68E-12	5.87	0.0084	303.9	315.8	92.5	98.3	0.105	0.135
M&F	Esophagus	2.27E-14	6.64	0.0099	24.6	27.0	82.5	85.4	0.010	0.009
M&F	[1] Hodgkin Lymphoma	2.69E-10	4.01	0.0092	4.3	3.8	77.5	81.9	0.003	0.002
M&F	Kidney and Renal Pelvis	5.55E-12	5.61	0.0099	66.4	68.9	77.5	82.7	0.029	0.026
M&F	Larynx	1.34E-12	5.62	0.0102	15.5	15.4	77.5	80.8	0.006	0.006
M&F	Leukemia	4.94E-15	7.19	0.0094	93.8	96.2	92.5	91.4	0.035	0.032
M&F	Liver and Intrahepatic Bile Duct	5.20E-11	4.97	0.0098	39.6	39.8	82.5	81.4	0.016	0.017
M&F	Lung and Bronchus	3.69E-15	7.68	0.0100	406.8	425.9	82.5	86.8	0.131	0.124
M&F	Melanoma of the Skin	5.96E-12	5.62	0.0092	108.8	109.8	92.5	89.1	0.045	0.044
M&F	Myeloma	3.67E-15	7.17	0.0099	44.3	46.0	82.5	86.6	0.015	0.014
M&F	Non-Hodgkin Lymphoma	4.43E-14	6.80	0.0097	115.9	120.5	82.5	87.9	0.042	0.040
M&F	Oral Cavity and Pharynx	7.25E-10	4.35	0.0092	47.5	45.4	92.5	83.3	0.022	0.022
M&F	Pancreas	1.83E-15	7.46	0.0095	107.7	111.0	92.5	91.0	0.036	0.035
M&F	Stomach	1.05E-14	6.90	0.0094	53.4	55.2	92.5	91.1	0.020	0.019
M&F	Thyroid	1.06E-06	2.56	0.0100	27.3	25.4	67.5	60.8	0.015	0.015
M&F	Urinary Bladder	3.08E-17	8.54	0.0098	181.0	201.9	87.5	90.3	0.059	0.055

[1]Hodgkin's lymphoma has a bimodal, rather than a unimodal distribution, thus does not well fit the multistage-senescence model.

Supplementary Table 5A. Male (M) parameter values of fits to SEER 2000-2003 age-specific cancer incidence rates employing the multistage-senescence model of Equation 4 (Pompei and Wilson, 2001; Harding et al. 2008).

Sex	Cancer type	Model -fitted			Peak incidence rate (per 100,000 person-year)		Age of peak incidence (yr)		Cumulative probability over lifespan	
		a	*k*	*b* (yr^{-1})	SEER	Model-fitted	SEER	Model-fitted	SEER	Model-fitted
M	All Major	5.29E-12	6.45	0.0093	3172.4	3755.9	87.5	90.5	1.236	1.353
M	All Major Non Sex	2.23E-12	6.55	0.0092	2333.2	2591.7	87.5	91.6	0.844	0.931
M	All Sites	5.44E-12	6.45	0.0093	3462.7	4087.6	87.5	91.1	1.337	1.481
M	Brain and Other Nervous System	3.67E-12	5.49	0.0099	27.0	27.5	77.5	83.0	0.012	0.011
M	Colon and Rectum	3.74E-14	7.11	0.0094	520.6	522.4	92.5	91.9	0.164	0.174
M	Esophagus	6.44E-14	6.55	0.0100	50.2	49.1	102.5	84.9	0.019	0.016
M	[1] Hodgkin Lymphoma	3.13E-10	4.07	0.0098	7.7	4.7	97.5	77.0	0.003	0.002
M	Kidney and Renal Pelvis	2.69E-12	5.82	0.0099	82.0	86.3	87.5	83.5	0.035	0.032
M	Larynx	1.17E-12	5.84	0.0102	33.4	35.4	72.5	81.2	0.013	0.013
M	Leukemia	3.38E-15	7.34	0.0093	136.0	138.5	92.5	92.8	0.046	0.045
M	Liver and Intrahepatic Bile Duct	2.21E-11	5.16	0.0095	43.3	44.3	77.5	84.5	0.018	0.018
M	Lung and Bronchus	1.60E-14	7.43	0.0099	563.9	644.4	77.5	87.4	0.198	0.196
M	Melanoma of the Skin	2.48E-11	5.29	0.0089	126.6	124.8	92.5	91.6	0.050	0.055
M	Myeloma	2.38E-15	7.29	0.0098	54.2	56.0	87.5	88.3	0.019	0.018
M	Non-Hodgkin Lymphoma	2.13E-13	6.45	0.0094	149.9	142.7	87.5	89.5	0.051	0.051
M	Oral Cavity and Pharynx	1.19E-09	4.32	0.0092	69.8	65.0	92.5	83.1	0.031	0.032
M	Pancreas	3.48E-15	7.33	0.0096	100.1	106.7	92.5	89.6	0.035	0.034
M	Stomach	2.01E-15	7.40	0.0094	120.4	101.8	102.5	92.0	0.036	0.033
M	Thyroid	1.80E-09	3.87	0.0102	10.9	10.2	67.5	72.4	0.005	0.005
M	Urinary Bladder	2.18E-16	8.21	0.0095	349.7	393.1	87.5	92.2	0.108	0.114
M	Prostate	2.90E-10	5.52	0.0118	1034.0	1107.3	72.5	84.5	0.388	0.356
M	Testis	3.11E-05	1.66	0.0237	12.9	12.0	32.5	31.7	0.004	0.003

[1]Hodgkin's lymphoma has a bimodal, rather than a unimodal distribution, thus does not well fit the multistage-senescence model.

The peak age-specific incidence rate, age of peak incidence, and cumulative probability over life span are computed up to maximum age 105, based on the SEER data alternatively model-fitted *ASR*(*t*).

Supplementary Table 5B. Female (F) parameter values of fits to SEER 2000-2003 age-specific cancer incidence rates employing the multistage-senescence model of Equation 4 (Pompei and Wilson, 2001; Harding et al. 2008).

Sex	Cancer type	Model -fitted			Peak incidence rate (per 100,000 person-year)		Age of peak incidence (yr)		Cumulative probability over lifespan	
		a	*k*	*b* (yr^{-1})	SEER	Model-fitted	SEER	Model-fitted	SEER	Model-fitted
F	All Major	5.28E-10	5.26	0.0093	1801.6	1861.2	87.5	87.5	0.771	0.789
F	All Major Non Sex	3.68E-12	6.30	0.0093	1262.8	1333.5	87.5	90.2	0.481	0.490
F	All Sites	4.53E-10	5.31	0.0092	2020.8	2074.8	87.5	88.5	0.853	0.882
F	Brain and Other Nervous System	6.62E-12	5.25	0.0098	18.8	17.8	77.5	82.6	0.008	0.007
F	Colon and Rectum	1.91E-14	7.18	0.0092	408.6	415.3	92.5	93.9	0.129	0.140
F	Esophagus	4.98E-16	7.34	0.0096	16.6	16.4	97.5	89.7	0.006	0.005
F	[1]Hodgkin Lymphoma	1.51E-11	4.67	0.0097	4.5	3.3	22.5	81.4	0.002	0.001
F	Kidney and Renal Pelvis	1.90E-12	5.73	0.0098	41.6	42.3	82.5	84.1	0.016	0.016
F	Larynx	6.35E-12	5.05	0.0104	6.7	5.4	72.5	76.9	0.002	0.002
F	Leukemia	8.77E-15	6.97	0.0091	75.0	75.5	97.5	94.1	0.026	0.026
F	Liver and Intrahepatic Bile Duct	3.37E-15	7.02	0.0098	23.2	22.9	102.5	87.4	0.009	0.007
F	Lung and Bronchus	2.33E-13	6.69	0.0100	325.5	324.0	77.5	84.9	0.110	0.106
F	Melanoma of the Skin	3.88E-08	3.31	0.0074	42.9	43.1	87.5	94.4	0.024	0.031
F	Myeloma	7.30E-15	6.93	0.0099	32.9	33.3	87.5	86.8	0.010	0.011
F	Non-Hodgkin Lymphoma	1.58E-13	6.45	0.0096	99.2	98.0	87.5	87.8	0.036	0.034
F	Oral Cavity and Pharynx	5.98E-12	5.34	0.0091	46.5	32.7	102.5	89.6	0.015	0.014
F	Pancreas	3.28E-16	7.81	0.0095	100.1	99.8	92.5	92.1	0.031	0.030
F	Stomach	6.67E-16	7.46	0.0091	56.3	56.1	92.5	95.5	0.017	0.019
F	Thyroid	5.26E-06	2.10	0.0099	20.3	19.7	52.5	52.9	0.012	0.013
F	Urinary Bladder	5.19E-16	7.68	0.0095	81.4	87.3	87.5	91.8	0.028	0.027
F	Breast	8.29E-07	3.35	0.0109	458.3	449.7	77.5	79.4	0.218	0.218
F	Cervix Uteri	3.31E-05	1.53	0.0087	16.4	15.3	42.5	54.8	0.011	0.012
F	Corpus Uteri	5.53E-08	3.65	0.0117	85.2	86.5	67.5	77.1	0.037	0.037
F	Ovary	6.60E-09	4.02	0.0110	58.7	57.4	82.5	83.4	0.025	0.025

[1]Hodgkin's lymphoma has a bimodal, rather than a unimodal distribution, thus does not well fit the multistage-senescence model.

Supplementary Table 5C. Male and female pooled (M&F) parameter values of fits to SEER 2000-2003 age-specific cancer incidence rates employing the multistage-senescence model of Equation 4 (Pompei and Wilson, 2001; Harding et al. 2008).

Sex	Cancer type	Model -fitted			Peak incidence rate (per 100,000 person-year)		Age of peak incidence (yr)		Cumulative probability over lifespan	
		a	*k*	*b* (yr^{-1})	SEER	Model-fitted	SEER	Model-fitted	SEER	Model-fitted
M&F	All Major	9.45E-11	5.71	0.0093	2254.2	2522.2	87.5	88.9	0.933	1.003
M&F	All Major Non Sex	7.20E-12	6.20	0.0093	1614.0	1764.7	87.5	90.7	0.610	0.661
M&F	All Sites	8.77E-11	5.74	0.0092	2494.1	2775.2	87.5	89.7	1.021	1.107
M&F	Brain and Other Nervous System	7.17E-12	5.28	0.0099	22.2	21.6	77.5	82.3	0.010	0.009
M&F	Colon and Rectum	5.30E-14	6.98	0.0092	438.3	452.9	87.5	93.1	0.143	0.156
M&F	Esophagus	8.33E-14	6.35	0.0099	25.5	28.0	87.5	85.2	0.010	0.010
M&F	[1] Hodgkin Lymphoma	1.55E-10	4.17	0.0097	4.3	3.8	27.5	78.6	0.003	0.002
M&F	Kidney and Renal Pelvis	5.19E-12	5.59	0.0099	56.9	59.1	77.5	83.0	0.023	0.022
M&F	Larynx	6.89E-12	5.28	0.0103	18.6	17.1	72.5	79.0	0.006	0.007
M&F	Leukemia	8.67E-15	7.05	0.0093	90.5	94.8	87.5	92.1	0.033	0.032
M&F	Liver and Intrahepatic Bile Duct	3.58E-12	5.50	0.0097	28.9	30.3	77.5	84.6	0.012	0.012
M&F	Lung and Bronchus	1.41E-13	6.85	0.0099	424.1	431.6	77.5	86.0	0.141	0.139
M&F	Melanoma of the Skin	2.57E-10	4.67	0.0091	69.8	69.4	82.5	86.3	0.033	0.033
M&F	Myeloma	5.48E-15	7.05	0.0099	39.9	41.2	87.5	86.9	0.013	0.013
M&F	Non-Hodgkin Lymphoma	2.43E-13	6.39	0.0096	115.8	113.0	87.5	87.9	0.042	0.040
M&F	Oral Cavity and Pharynx	4.56E-10	4.45	0.0093	45.1	44.1	102.5	83.8	0.021	0.021
M&F	Pancreas	1.98E-15	7.42	0.0095	100.0	102.1	92.5	91.4	0.033	0.032
M&F	Stomach	3.12E-15	7.21	0.0093	67.9	69.5	92.5	92.3	0.023	0.023
M&F	Thyroid	6.51E-07	2.53	0.0098	14.7	14.3	67.5	62.0	0.009	0.009
M&F	Urinary Bladder	1.24E-15	7.68	0.0096	169.3	186.3	87.5	90.5	0.055	0.057

[1]Hodgkin's lymphoma has a bimodal, rather than a unimodal distribution, thus does not well fit the multistage-senescence model.
