# Supplementary material for: Profound synchrony of age-specific incidence rates and tumor suppression for different cancer types as revealed by the multistage-senescence model of carcinogenesis
Source: Aging (Albany NY). 2021 Oct 25;13(20):23545–78. doi: 10.18632/aging.203651 (PMC8580351; doi:10.18632/aging.203651)
Supplement: Supplementary Notes [file aging-13-203651-s003.docx]

**Supplementary Notes**

**Note 1. Comparison of *k* and *b* for reproductive and non-reproductive cancers**

*2010–2013 Data*

The issue of multiple comparisons occurs when testing multiple hypotheses based on one set of data. The threshold for a hypothesis test to be considered statistically significant is 10%. All *P* values are given for two-sided *t* tests. The hypotheses and null hypotheses (H_0_) are listed below.

(1)^+^ *b*_male, non-reproductive_ > *b*_female, non-reproductive_ (H_0_: *b*_male, non-reproductive_ = *b*_female, non-reproductive_), *p* value: 0.03417

(2)^♦^ *b*_female, reproductive_ > *b*_female, non-reproductive_ (H_0_: *b*_female, reproductive_ = *b*_female, non-reproductive_), *p* value: 0.00208

(3) *b*_male & female, reproductive_ > *b*_male & female, non-reproductive_ (H_0_: *b*_male & female, reproductive_ = *b*_male & female, non-reproductive_), *p* value: 0.0736

(4)^+^ *k*_male, non-reproductive_ > *k*_female, non-reproductive_ (H_0_: *k*_male, non-reproductive_ = *k*_female, non-reproductive_), *p* value: 0.003306

(5) *k*_female, non-reproductive_ > *k*_female, reproductive_ (H_0_: *k*_female, reproductive_ = *k*_female, non-reproductive_), *p* value: 0.01934

(6) *k*_male & female, non-reproductive_ > *k*_male & female, reproductive_ (H_0_: *k*_male & female, reproductive_ = *k*_male & female, non-reproductive_), *p* value: 0.002686

Now, if one test is performed at the 10% level and if the corresponding null hypothesis is true, there is only a 10% chance of incorrectly rejecting the null hypothesis. However, in our case, with six tests the probability of incorrectly rejecting at least one null hypothesis now becomes 1 – (1 – 0.1)^6^ = 47%, which is well above the prescribed 10%.

To prevent this problem, we implement the Holm method.

Rank of *p* value: 0.00208, 0.002686, 0.003306, 0.01934, 0.03417, and 0.0736

Threshold: 0.1/6, 0.1/5, 0.1/4, 0.1/3, 0.1/2, and 0.1/1

As all *p* value are smaller than their respective thresholds, all six null hypotheses are rejected.

^+^ To compare difference between male and female for non-reproductive cancers, READ has been left out because it applies to only males.
^♦^ *b*_male, reproductive_ > *b*_male, non-reproductive_ were not compared, as there are only two male reproductive cancers.

*2000–2003 Data*

Now, we test the replicability of the above results in the *2000–2003* set of data.

(1)^*+^ *b*_male, non-reproductive_ > *b*_female, non-reproductive_ (H_0_: *b*_male, non-reproductive_ = *b*_female, non-reproductive_), *p* value: 0.02096

(2) *b*_female, reproductive_ > *b*_female, non-reproductive_ (H_0_*: b*_female, reproductive_ = *b*_female, non-reproductive_), *p* value: 0.01807

(3) *b*_male & female, reproductive_ > *b*_male & female, non-reproductive_ (H_0_: *b*_male & female, reproductive_ = *b*_male & female, non-reproductive_), *p* value: 0.1103

(4)^+^ *k*_male, non-reproductive_ > *k*_female, non-reproductive_ (H_0_: *k*_male, non-reproductive_ = *k*_female, non-reproductive_), *p* value: 0.02694

(5)^*^ *k*_female, non-reproductive_ > *k*_female, reproductive_ (H_0_: *k*_female, reproductive_ = *k*_female, non-reproductive_), *p* value: 0.005146

(6) *k*_male & female, non-reproductive_ > *k*_male & female, reproductive_ (H_0_: *k*_male & female, reproductive_ = *k*_male & female, non-reproductive_), *p* value: 0.001719

^*^There may be considerable deviation from a normal distribution, unlike in the 2010–2013 data.

^+^ACC and SARC have been left out as they apply to only male.

Rank of *p* values: 0.001719, 0.005146, 0.01807, 0.02096, 0.02694, and 0.1103

Threshold: 0.1/6, 0.1/5, 0.1/4, 0.1/3, 0.1/2, and 0.1/1

We only fail to reject test (3) but we reject every other null hypothesis.

As far as replicability goes, we suggest that there is no difference between the suppressing factors for reproductive versus non-reproductive cancers when considering both sexes together, test (3), and that the differences are indeed significant for the other tests.

**Note 2. ANOVA comparing the male and female lines of *u* regressed against *k***

For male: *u* = 0.0042*k* – 0.0044 Model (1)

For female: *u* = 0.0038*k* – 0.0048 Model (2)

As the linear trends are very similar, we consider if there is any statistical difference. As these two models result from two non-overlapping data sets, they cannot be directly compared with ANOVA or ANCOVA. Instead, they were combined into one large data set and *u* regressed against *k* and sex. Now, if sex as a factor turns out to be insignificant, we can conclude that there is in fact negligible difference. We provide the R code below for performing this step, where allgfits_u is our data frame containing the information of Table 1A, 1B, as we use only the M and F data, and not the “both” (M&F) data.

*Two-variable model (1)*

model1A <- lm(uu ~ kk + as.factor(sex), allgfits_u[allgfits_u$sex != 'both',])

> summary(model1A)

Call: lm(formula = uu ~ kk + as.factor(sex), data = allgfits_u[allgfits_u$sex != "both", ])

**Table A. Coefficients**

|  | **Estimate** | **Standard error** | ***t* value** | **Pr(>\|t\|)** |
| --- | --- | --- | --- | --- |
| **Intercept** | -0.0058359 | 0.0027693 | -2.107 | 0.0407 * |
| **kk** | 0.0040289 | 0.0004593 | 8.772 | 2.67e-11 *** |
| **as.factor(sex)male** | 0.0024398 | 0.0017163 | 1.422 | 0.1621 |

Significance codes: 0 ‘***’; 0.001 ‘**’; 0.01 ‘*’; 0.05 ‘.’; 0.1 ‘ ’ ;1

Residual standard error: 0.005773 on 45 degrees of freedom

Multiple *R*-squared: 0.6663, Adjusted *R*-squared: 0.6514, *F*-statistic: 44.92 on 2 and 45 DF, *p* value: 1.889e-11

We see that sex has a *p* value above 10%, so we opt to drop it and see what happens.

*Two-variable Model (2)*

model2A <- lm(uu ~ kk, allgfits_u[allgfits_u$sex != 'both',])

Call: lm(formula = uu ~ kk, data = allgfits_u[allgfits_u$sex != "both", ])

**Table B. Residuals**

| **Min** | **1Q** | **Median** | **3Q** | **Max** |
| --- | --- | --- | --- | --- |
| -0.0104201 | -0.0039084 | 0.0002108 | 0.0023458 | 0.0187606 |

**Table C. Coefficients**

|  | **Estimate** | **Standard error** | ***t* value** | **Pr(>\|t\|)** |
| --- | --- | --- | --- | --- |
| **Intercept** | -0.0055757 | 0.0027937 | -1.996 | 0.0519 |
| **kk** | 0.0041829 | 0.0004513 | 9.269 | 4.3e-12 *** |

Significance codes: 0 ‘***’; 0.001 ‘**’; 0.01 ‘*’; 0.05 ‘.’; 0.1 ‘ ’ ;1

Residual standard error: 0.005837 on 46 degrees of freedom

Multiple *R*-squared: 0.6513, Adjusted *R*-squared: 0.6437, *F*-statistic: 85.91 on 1 and 46 DF, *p* value: 4.299e-12

*ANOVA*

Now that we are using the same dataset for both models and that Model (2) is “nested” in Model (1) [as we dropped one variable from Model (1) to make Model (2)], we can use ANOVA to double-check if the linear trends are statistically different:

> anova(model2A, model1A) # *p* value = 0.1621

**Table D. Analysis of variance for Model (1): uu ~ kk and Model (2): uu ~ kk + as.factor(sex)**

| **Model** | **Res.Df** | **RSS** | **Df** | **Sum of Sq** | **F-statistic** | **Pr(>F)** |
| --- | --- | --- | --- | --- | --- | --- |
| **(1)** | 46 | 0.0015670 |  |  |  |  |
| **(2)** | 45 | 0.0014996 | 1 | 6.734e-05 | 2.0207 | 0.1621 |

Indeed, sex can be omitted from the model and can conclude that the slope and intercept of *u* versus *k* are the same for both male and female cancers.

*Final Two-variable Model*

Employing the methodology explained above, we now develop a two-variable model equation based on M and F data given in Table 1A, 1B and shown in Figure 1A, 1B in the manuscript, specifically, when a plot that takes the data shown in Figure 2A, 2B and puts them together as one line in a statistically sound way. This is not employed in Equation 6, as this employs data for both males and females that are pooled (M&F, Table 1C), hence are not separate as here.

For male & female, *u* = 0.0042*k* – 0.0056.

The average of the male and female slopes is not appropriate because the 2010–2013 data for males and females have different variances (male: 1.065 versus female: 0.717), almost by a factor of 1.5. The slope of the best-fit line is influenced by the variance in the data. As the male component of the overall data has a much larger variance, it has a greater influence in where the trend line should go.

Analogously, for the 2000–2003 data, the line of best fit for male and female is *u* = 0.0043*k* – 0.0062.

**Note 3. Computation of increase in cumulative probability over a lifetime, exponential versus multistage-senescence model**

This note explains the computation for cancer stages *k* = 2, 3, 4, 6, and 8 the cumulative probability up to 101 years (=1/*b*) being reduced by 67, 75, 80, 85, and 88%, respectively, indicating greater senescence-tumor suppression *b* for the more complex cancers of longer latent periods.

We estimate *k* first, then *u* from *k*. Then we take average of *b* and apply the formula. We do need to assume that the *k* (and hence *u*) estimates from the power function are in close range with those from the beta model. In this case, *u* = 0.0046*k* – 0.0087. Reminder: The quantities below describe the probability of getting cancer in one’s lifetime, one according to beta (multistage-senescence) model and the other according to the Armitage and Doll model (1954).

Beta: $\int_{0}^{\frac{1}{b}} at^{k-1}\left( 1-bt \right)dt=\frac{a}{b^{k}}\left( \frac{1}{k}-\frac{1}{k+1} \right)=\frac{a}{b^{k}k(k+1)}$ Equation S1)

Power: $1- e^{-\int_{0}^{\frac{1}{b}} at^{k-1}dt}=1-e^{-\frac{a}{b^{k}k}}$ Equation S2)

Reduction rate = 1 – (Equation S1)/(Equation S2).

Where: *a* = $\frac{u^{k}}{\Gamma(k)}$.
